# Performance of the Cas9 Nickase System in *Drosophila melanogaster*

**DOI:** 10.1534/g3.114.013821

**Published:** 2014-08-15

**Authors:** Xingjie Ren, Zhihao Yang, Decai Mao, Zai Chang, Huan-Huan Qiao, Xia Wang, Jin Sun, Qun Hu, Yan Cui, Lu-Ping Liu, Jun-Yuan Ji, Jiang Xu, Jian-Quan Ni

**Affiliations:** * Gene Regulatory Lab, School of Medicine, Tsinghua University, Beijing 100084, China; † School of Life Sciences, Tsinghua University, Beijing 100084, China; ‡ Tsinghua Fly Center, Tsinghua University, Beijing 100084, China; § Department of Molecular and Cellular Medicine, College of Medicine, Texas A&M Health Science Center, College Station, Texas 77843; ** School of Basic Medical Sciences, Wuhan University, Wuhan 430071, China; †† College of Bioengineering, Hubei University of Technology, Wuhan 430068, China

**Keywords:** CRISPR, Cas9, off-target, nickase, *piwi*

## Abstract

Recent studies of the Cas9/sgRNA system in *Drosophila melanogaster* genome editing have opened new opportunities to generate site-specific mutant collections in a high-throughput manner. However, off-target effects of the system are still a major concern when analyzing mutant phenotypes. Mutations converting Cas9 to a DNA nickase have great potential for reducing off-target effects *in vitro*. Here, we demonstrated that injection of two plasmids encoding neighboring offset sgRNAs into transgenic Cas9^D10A^ nickase flies efficiently produces heritable indel mutants. We then determined the effective distance between the two sgRNA targets and their orientations that affected the ability of the sgRNA pairs to generate mutations when expressed in the transgenic nickase flies. Interestingly, Cas9 nickase greatly reduces the ability to generate mutants with one sgRNA, suggesting that the application of Cas9 nickase and sgRNA pairs can almost avoid off-target effects when generating indel mutants. Finally, a defined *piwi* mutant allele is generated with this system through homology-directed repair. However, Cas9^D10A^ is not as effective as Cas9 in replacing the entire coding sequence of *piwi* with two sgRNAs.

Recent studies of the CRISPR (clustered regularly interspaced short palindromic repeat) system have broad applications in genome editing (Deltcheva *et al.* 2011; Gasiunas *et al.* 2012; Jinek *et al.* 2012; Cong *et al.* 2013; Mali *et al.* 2013b). The CRISPR system provides adaptive immunity against invading viruses and plasmids in bacteria and archaea (Deveau *et al.* 2010; Bhaya *et al.* 2011; Terns and Terns 2011; Wiedenheft *et al.* 2012). In the type II CRISPR system, transcript from the CRISPR array is first processed into small CRISPR RNA (crRNA). Together with *trans*-encoded tracrRNA, crRNA then guides CRISPR-associated protein 9 (Cas9) to cleave foreign double-stranded DNA with sequence specificity provided by base-pairings between the crRNA and the target DNA. It has been shown that type II *Streptococcus pyogenes* Cas9, when guided by a single-guide-RNA (sgRNA), a crRNA-tracrRNA chimera, can generate DNA double-stranded breaks (DSBs) *in vitro* (Jinek *et al.* 2012; Cong *et al.* 2013; Mali *et al.* 2013b). In addition, a three-nucleotide (NGG) protospacer adjacent motif (PAM) sequence in the DNA is required for the *S. pyogenes* Cas9/sgRNA system to target and cleave the double strand.

The Cas9/sgRNA system has been successfully applied in *Drosophila melanogaster* to generate DSBs in the genome and induce indel mutations through nonhomologous end joining (NHEJ) or sequence-specific mutations through homology-directed repairs (HDRs) for recessive viable genes (Baena-Lopez *et al.* 2013; Bassett *et al.* 2013; Gratz *et al.* 2013; Kondo and Ueda 2013; Ren *et al.* 2013; Sebo *et al.* 2013; Yu *et al.* 2013; Gratz *et al.* 2014; Xue *et al.* 2014; Yu *et al.* 2014). However, potential off-target DSBs might result in unexpected indel mutations, especially when relying on NHEJ, thus increasing the complexity of analyzing mutants of interest (Fu *et al.* 2013; Hsu *et al.* 2013; Cho *et al.* 2014). Cas9 has a RuvC nuclease domain that targets the DNA strand noncomplementary to the sgRNA and a HNH nuclease domain that targets the complementary strand (Supporting Information, Figure S1A), and mutations in either one of the two domains convert Cas9 into a DNA nickase (Jinek *et al.* 2014; Nishimasu *et al.* 2014). Previous reports showed that Cas9 nickase with a pair of offset sgRNAs that target opposite strands of DNA are capable of inducing DSBs *in vitro* while almost avoiding off-target DSBs (Mali *et al.* 2013a; Ran *et al.* 2013; Cho *et al.* 2014). In addition, coupled with one sgRNA, Cas9 nickase has been used to generate sequence-specific mutations through the HDR pathway *in vitro* (Cong *et al.* 2013; Hsu *et al.* 2013; Ran *et al.* 2013). However, less is known regarding the performance of Cas9 nickase with a pair of sgRNAs in replacing entire coding sequences of genes through HDR *in vivo*.

Here, we developed transgenic flies that specifically express Cas9 nickase in the germline via the *nanos* regulatory sequence. We then tested the indel mutation rate of Cas9 nickase with paired DNA constructs of sgRNAs and found that neighboring sgRNAs −1 to 26 bp apart that supposedly leave a 5′ overhang can efficiently generate DSBs and induce mutations. In addition, we have observed that one sgRNA does not trigger mutations in Cas9 nickase transgenic flies, suggesting that the Cas9 nickase system significantly prevents off-target effects. However, Cas9^D10A^ is not as effective as Cas9 nuclease in replacing the entire coding sequence of essential genes such as *piwi* using two sgRNAs through HDR.

## Materials and Methods

### sgRNA and *nos*-Cas9^D10A^ vector construct

sgRNAs were designed using the online CRISPR design tool (http://www.flyrnai.org/crispr/) and were cloned into the *U6b*-sgRNA-short vector as previously described (Ren *et al.* 2013). To express Cas9 nickases in *Drosophila* germ cells, we utilized a previously constructed *nos*-Cas9 plasmid (Ren *et al.* 2013) containing wild-type Cas9, approximately 700 base pairs of the *nos* promoter, the *nos* 5′UTR, and the *nos* 3′UTR. The *attB* donor sequence and a wild-type *vermillion^+^* marker were also included in the *nos*-Cas9 plasmid. The Cas9^D10A^ gene was generated by converting the tenth GAT codon into GCT using the AccuPrime Pfx DNA Polymerase Kit (Invitrogen) according to the manufacturer’s instructions. The Cas9^H840A^ gene was constructed in the same way by converting the 840^th^ CAT codon into GCT. The sequences of the *nos*-Cas9^D10A^ and *nos*-Cas9^H840A^ plasmids are shown in Figure S2. The oligonucleotides used for cloning are listed in Table S1.

### Donor vector construct for HDR at the *piwi* locus

The piwi-4XP3-mCherry donor construct was based on the pBluescript plasmid. The left homologous arm of *piwi* was amplified from genomic extract with primers piwi-L-F and piwi-L-R and was cloned into the *Avr*II and *Nhe*I sites. The right homologous arm was amplified with primers piwi-R-F and piwi-R-R and was cloned into the *Bam*HI and *Spe*I sites. The selection marker 4XP3-mCherry was constructed on a different pBluescript vector first. The gene encoding the red fluorescent protein mCherry was amplified with primers mCherry-F and mCherry-R and was cloned into the *Xho*I and *Kpn*I sites. The 4XP3 promoter sequence (Horn *et al.* 2000) was synthesized and cloned into the *Hin*dIII and *Xho*I sites. The SV40 3′UTR sequence was amplified with primers SV40-F and SV40-R and was cloned into the *Kpn*I and *Eco*RV sites. The selection marker 4XP3-mCherry was then cut and inserted between the left and right homologous arms of *piwi* to finish the piwi-4XP3-mCherry construct. All PCR fragments were amplified with *pfu* DNA polymerase (TransGen Biotech, Beijing). The donor construct was confirmed by sequencing (Invitrogen, Beijing).

### DNA purification and embryo injection

DNA plasmid solution was thoroughly mixed with 1/10 volume of 3 M sodium acetate (pH 5.2, AMRESCO) and 5 volumes of absolute ethanol, stored at −20° for 2 hr, and centrifuged at 21,000×*g*. The DNA pellet was washed twice in 70% ethanol, twice in 100% ethanol, and re-suspended in injection buffer for the appropriate concentration. The *Drosophila* embryos were injected using previously described protocols (Ren *et al.* 2013). The injection concentrations of sgRNA plasmids were at 100 ng/µL when one sgRNA was used and at 100 ng/µL each when two sgRNAs were used. The concentration of the donor template for HDR was at 400 ng/µL.

### Fly stocks and mutation screening

All flies were cultured on standard cornmeal food at 25°. The *P{nos-Cas9^D10A^}attP2* stock was previously established (Ren *et al.* 2013). The *P{nos-Cas9^D10A^}attP40*, *P{nos-Cas9^D10A^}attP2*, *P{nos-Cas9^H840A^}attP40*, and *P{nos-Cas9^H840A^}attP2* fly stocks were established according to a previously described protocol (Ni *et al.* 2008).

To score for germline mutations, G0 adult flies that developed from injected *y[1] sc[1] v[1]*;; *P{nos-Cas9^D10A^}attP2* or *y[1] sc[1] v[1]*;; *P{nos-Cas9^H840A^}attP2* embryos were crossed to *y[1] w[67c23]* for *white* mutations. The F1 progeny were screened for the first 6 d after eclosion. The heritable mutation rate was calculated as the number of all mutant F1 progeny divided by the number of all progeny screened for a given sgRNA target. Successful *piwi^HDR-mCherry^* mutants were screened by the expression of mCherry in the eyes under a Leica MZ10F fluorescent microscope. Mutagenesis events were confirmed by sequencing of F1 adults, and the detection primers are listed in Table S1.

### Genomic DNA extraction

Fly genomic DNA was purified via phenol-chloroform extraction. Single flies were homogenized in 400 μL of lysis buffer (1× PBS, 0.2% SDS, and 200 μg/mL proteinase K) and incubated at 50° for 1 hr, followed by extraction in 400 μL of phenol-chloroform. The mixture was then centrifuged at 21,000×*g* for 20 min at 4°, and the supernatant was then transferred to a new tube. An equal volume of isopropanol was added, and the tube was vortexed thoroughly. The mixture was then kept at −20° for at least 1 hr, followed by centrifugation at 21,000×*g* for 20 min at 4°. The supernatant was removed, and the DNA pellet was washed with 500 μL of 75% ethanol, followed by centrifugation at 21,000×*g* for 5 min at 4°. Finally, the pellet was dried for 10 min and re-suspended in 30 μL of DNase-free water.

### Off-target analysis

To investigate the possibility of off-target cleavage by Cas9 nickase/sgRNA, we searched the fly genome for potential off-targets containing no more than four mismatches to the on-target, followed by a PAM sequence. Primers flanking the potential off-targets were used to PCR-amplify these regions for analysis by sequencing. For sequencing analysis, genomic DNA from a single fly was used as template, and the defined DNA fragment was amplified by specific primers (Table S2).

### Immunostaining of ovaries

Ovaries were dissected in cold PBS and fixed in PBS with 4% paraformaldehyde for 15 min, then washed with PBT (PBS and 0.3% Triton X-100) five times for 15 min each. The ovaries were incubated in 0.5% goat serum diluted in PBT for 1 hr. Appropriate primary antibodies were added to PBS and incubated at 4° overnight, then washed with PBT five times for 15 min each. Appropriate secondary antibodies were then added and incubated at 25° for 2 hr; they were washed with PBT five times for 15 min each. After the last wash, the stained ovaries were mounted in Fluoromount mounting media (F4680; Sigma). Images were obtained with an inverted Zeiss LSM780 fitted with a UV laser.

The following primary and secondary antibodies were used: mouse monoclonal anti-Hts antibody 1B1 (DSHB, 1:100); rabbit anti-Piwi (1:200; Santa Cruz sc-98264); FITC-conjugated anti-mouse IgG (1:200; Jackson ImmunoResearch); and TRITC-conjugated anti-rabbit IgG (1:200; Jackson ImmunoResearch).

## Results

### Generating Cas9 nickase transgenic fly lines

**Figure 1:**
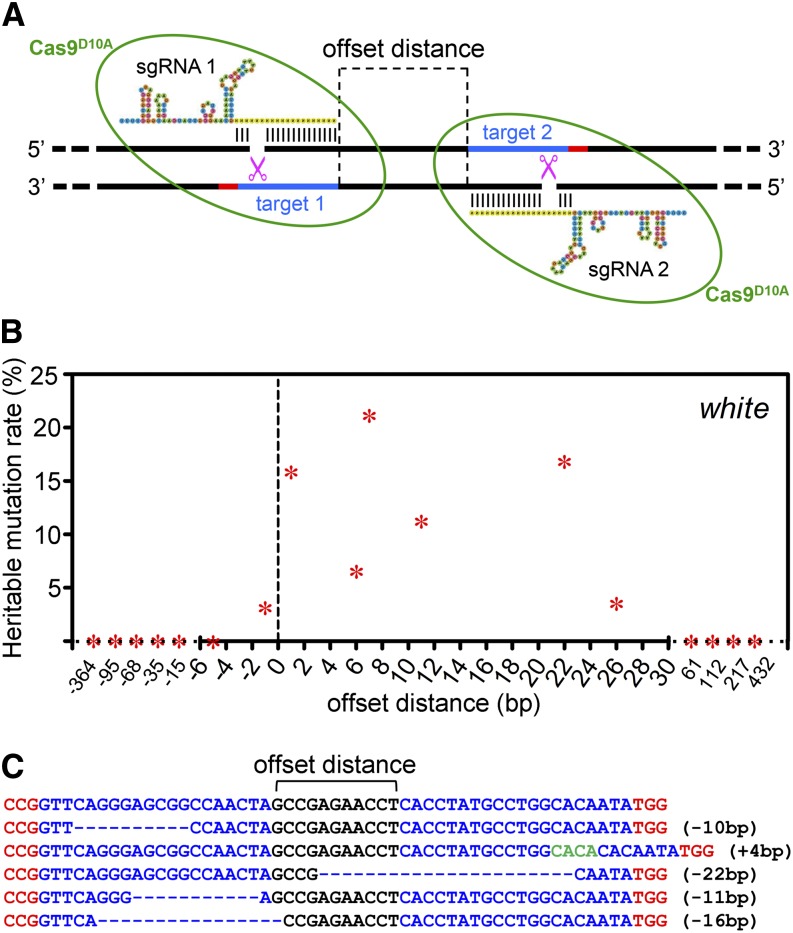
A pair of sgRNAs targeting close regions in the genome can introduce indel mutations in transgenic Cas9^D10A^ nickase flies. (A) Diagram showing two neighboring Cas9^D10A^/sgRNA ribonucleoproteins targeting the fly genome. The D10A mutation converts Cas9 into a nickase that targets only the strand complementary to the sgRNA. Each Cas9^D10A^ nickase is shown as a green circle. The sgRNA targets are shown in blue and the PAMs are shown in red. The single-strand cutting sites are shown by the magenta scissors. The offset distance is measured from the PAM-distal end of an sgRNA target to that of the other. If the PAMs are facing outward away from each other as shown in this diagram, then the distance is a positive number. (B) Scatter plot showing the relationship between heritable *white* mutation rate and sgRNA distance when using offset pairs in the Cas9^D10A^ transgenic flies. Heritable mutation rate is calculated as number of the white-eyed mutant F1 flies divided by total F1 flies screened. (C) Representative sequencing results showing mutations generated with Cas9^D10A^ transgenic flies and a pair of sgRNAs with offset distance of 11 bp. The sgRNA targets are in blue, and the PAMs are shown in red. Mutations with deleted nucleotides are shown with dashed lines, and inserted nucleotides are shown in green.

The Cas9/sgRNA system has been introduced into *Drosophila melanogaster* to generate heritable mutations. However, sgRNAs may induce off-target cuttings on DNA sequences other than the intended target due to tolerated mismatches between sgRNAs and DNA. To reduce or prevent off-target effects, we deactivated either the RuvC nuclease activity of Cas9 by converting the 10^th^ amino acid Asn (D) to Ala (A) (Figure 1A) or the HNH activity by changing the 840^th^ residue His (H) to Ala (A) (Figure S1B). We then integrated the Cas9^D10A^ transgene into an *attP2* site on chromosome 3, or into an *attP40* site on chromosome 2 (Ni *et al.* 2008). Cas9^H840A^ transgenic flies were generated in the same manner. Similar to wild-type control, transgenic Cas9^D10A^ flies and Cas9^H840A^ flies that specifically express Cas9 nickases in the germlines are healthy and fertile.

### Cas9 nickase efficiently generates heritable mutants when coupled with a pair of offset sgRNAs

To examine the mutagenesis efficiency of Cas9 nickase, we constructed a series of sgRNA vectors to target the *white* (*w*) gene and introduced pairs of them into *P{nos-Cas9^D10A^}attp2* embryos by co-injection (Figure 1B). We then crossed the injected G0s to *w* flies and evaluated the heritable mutation rates by screening for white-eyed F1 progeny.

Consistent with previous *in vitro* data from cell culture, only offset sgRNA pairs that supposedly leave a 5′ overhang after Cas9^D10A^ cleavage can generate mutations, and such pairs with an offset range (PAM-distal end of an sgRNA to that of the other) from −1 to 26 bp had the ability to produce a mutagenesis rate up to 21.2%, with an average mutation rate of 11.2% (Figure 1B, Table 1). In addition, almost no indel mutants were generated with sgRNA offset distance outside of this range (Figure 1B, Table 1). Interestingly, sgRNA pairs complementing the same DNA single strand did not generate any mutants. Taken together, these results show that the efficiency of heritable mutation is dependent on the proper distance as well as orientations between the two sgRNA targets.

**Table 1:** Heritable mutation rates of sgRNAs with the wild-type Cas9 or Cas9^D10A^ nickase

Gene Name (CG#)	sgRNA Offset (bp)*	sgRNA Name	sgRNA Target Sequence	Heritable Mutation Rate (%)**		
				With Cas9	With Cas9^D10A^	Both sgRNAs with Cas9^D10A^
*white* (CG2759)	−364	white-J	CTGCGGCGATCGAAAGGCAA	57.1	ND	0
		white-F	CGCCGGAGGACTCCGGTTCA	18.1	ND	
	−95	white-E	TAGTTGGCCGCTCCCTGAAC	32.3	0	0
		white-K	GCTGCATTAACCAGGGCTTC	64.1	ND	
	−68	white-E	TAGTTGGCCGCTCCCTGAAC	32.3	0	0
		white-L	CCAAAAACTACGGCACGCTC	78.6	0	
	−35	white-E	TAGTTGGCCGCTCCCTGAAC	32.3	0	0
		white-F	CGCCGGAGGACTCCGGTTCA	18.1	ND	
	−15	white-D	AGGTGAGGTTCTCGGCTAGT	57.1	<0.1%	0.2
		white-R	CCGAGAACCTCACCTATGCC	58.6	0	
	−5	white-D	AGGTGAGGTTCTCGGCTAGT	57.1	<0.1%	0
		white-H	CACCTATGCCTGGCACAATA	42.4	ND	
	−1	white-Q	GATCCTCTTGGCCCATTGCC	52.6	ND	3.2
		white-B	CAGGAGCTATTAATTCGCGG	61.8	<0.1%	
	1	white-E	TAGTTGGCCGCTCCCTGAAC	32.3	0	15.9
		white-R	CCGAGAACCTCACCTATGCC	58.6	0	
	6	white-D	AGGTGAGGTTCTCGGCTAGT	57.1	<0.1%	6.6
		white-I	GGCACAATATGGACATCTTT	52.1	0	
	7	white-D	AGGTGAGGTTCTCGGCTAGT	57.1	<0.1%	21.2
		white-C	GCACAATATGGACATCTTTG	43.7	0	
	11	white-E	TAGTTGGCCGCTCCCTGAAC	32.3	0	11.3
		white-H	CACCTATGCCTGGCACAATA	42.4	ND	
	22	white-E	TAGTTGGCCGCTCCCTGAAC	32.3	0	16.9
		white-I	GGCACAATATGGACATCTTT	52.1	0	
	26	white-E	TAGTTGGCCGCTCCCTGAAC	32.3	0	3.6
		white-A	CAATATGGACATCTTTGGGG	81.6	0	
	61	white-F	CGCCGGAGGACTCCGGTTCA	18.1	ND	0
		white-A	CAATATGGACATCTTTGGGG	81.6	0	
	112	white-E	TAGTTGGCCGCTCCCTGAAC	32.3	0	0
		white-G	AGCGACACATACCGGCGCCC	80.1	<0.1%	
	217	white-E	TAGTTGGCCGCTCCCTGAAC	32.3	0	0
		white-A	CAATATGGACATCTTTGGGG	81.6	0	
	432	white-E	TAGTTGGCCGCTCCCTGAAC	32.3	0	0
		white-O	TTATCGGCTCCCTAACGGCC	71.4	ND	

ND, Not done.

* Offset distance of a given sgRNA pair is measured from PAM-distal end of an sgRNA to that of the other. When the PAMs are facing outward relative to each other, the distance is a positive number. If the PAMs are facing inward toward each other, then the distance is a negative number.

** The heritable mutation rate is calculated as the number of white-eyed F1s divided by the number of all F1s observed.

To examine the extent of the indels, we randomly sequenced mutant flies from the group of offset sgRNA pairs with distance of 11 bp. From the sequencing results of six independent F1 mutants, we found that indel mutations occurred around the targeted region (Figure 1C).

To evaluate the Cas9^H840A^ nickase, we performed a similar mutagenesis test as described above. Unlike Cas9^D10A^, Cas9^H840A^ was designed to cut only the DNA strand that was not complementary to the sgRNA (Figure S1B). Thus, sgRNA pairs with an offset distance of less than −30 bp should leave a 5′ overhang (Figure S1B). Consistent with transgenic Cas9^D10A^ flies, only neighboring sgRNA pairs could trigger DSBs with Cas9^H840A^ flies (Table 2). However, the mutagenesis rates of Cas9^H840A^ flies were much lower compared with Cas9^D10A^ flies, reaching only up to 1.5% (Table 2).

**Table 2:** Heritable mutation rates of sgRNAs with the wild-type Cas9 or Cas9^H840A^ nickase

Gene Name (CG#)	sgRNA Offset (bp)*	sgRNA Name	sgRNA Target Sequence	Heritable Mutation Rate (%)**		
				With Cas9	With Cas9^H840A^	Both sgRNAs with Cas9^H840A^
*white* (CG2759)	−88	white-K	GCTGCATTAACCAGGGCTTC	64.1	ND	0.8
		white-S	GAGGACTCCGGTTCAGGGAG	78.5	0	
	−84	white-F	CGCCGGAGGACTCCGGTTCA	18.1	0	0.8
		white-L	CCAAAAACTACGGCACGCTC	78.6	0	
	−69	white-K	GCTGCATTAACCAGGGCTTC	64.1	ND	0.8
		white-P	CCTCCGGCGGACTGGGTGGC	80.2	0	
	−68	white-F	CGCCGGAGGACTCCGGTTCA	18.1	0	1.1
		white-M	GCTCCGGCCACCCAGTCCGC	63.6	0	
	−42	white-S	GAGGACTCCGGTTCAGGGAG	78.5	0	1.0
		white-N	CCGGCCACCCAGTCCGCCGG	33.2	0	
	−35	white-E	TAGTTGGCCGCTCCCTGAAC	32.3	0	1.5
		white-F	CGCCGGAGGACTCCGGTTCA	18.1	0	
	−15	white-D	AGGTGAGGTTCTCGGCTAGT	57.1	0	0
		white-R	CCGAGAACCTCACCTATGCC	58.6	0	
	−5	white-D	AGGTGAGGTTCTCGGCTAGT	57.1	0	0
		white-H	CACCTATGCCTGGCACAATA	42.4	ND	
	−1	white-Q	GATCCTCTTGGCCCATTGCC	52.6	ND	0
		white-B	CAGGAGCTATTAATTCGCGG	61.8	0	
	1	white-E	TAGTTGGCCGCTCCCTGAAC	32.3	0	0
		white-R	CCGAGAACCTCACCTATGCC	58.6	0	
	7	white-D	AGGTGAGGTTCTCGGCTAGT	57.1	0	0
		white-C	GCACAATATGGACATCTTTG	43.7	0	
	11	white-E	TAGTTGGCCGCTCCCTGAAC	32.3	0	0
		white-H	CACCTATGCCTGGCACAATA	42.4	ND	
	22	white-E	TAGTTGGCCGCTCCCTGAAC	32.3	0	0
		white-I	GGCACAATATGGACATCTTT	52.1	0	

ND, Not done.

* Offset distance of a given sgRNA pair is measured from PAM-distal end of an sgRNA to that of the other. When the PAMs are facing outward relative to each other, the distance is a positive number. If the PAMs are facing inward toward each other, then the distance is a negative number.

** The heritable mutation rate is calculated as the number of white-eyed F1s divided by the number of all F1s observed.

The survival and fertile G0 rates were evaluated for the Cas9^D10A^ and Cas9^H840A^ methods (Table S3) and compared with those of wild-type Cas9. We also focused on the survival and fertile G0 rates of sgRNA pairs that successfully generated heritable mutants with Cas9 nickase (Figure S3). However, no significant improvements in G0 survival and fertile rates were observed when applying either version of the Cas9 nickase with paired sgRNAs compared with Cas9 nuclease and one sgRNA (Figure S3).

### No off-target effects detected when using Cas9^D10A^ flies

The major purpose of applying the Cas9^D10A^ nickase is to reduce or prevent off-target effects associated with Cas9. Previous studies demonstrate that the numbers and positions of mismatches in nucleotides between the sgRNA and the DNA target affect the rate of off-target cuts, and as many as five mismatches could be tolerated for Cas9 to introduce DSBs *in vitro* (Fu *et al.* 2013; Hsu *et al.* 2013; Cho *et al.* 2014). To test whether the Cas9^D10A^/sgRNA limits off-target effects, we individually introduced the sgRNAs targeting *w* into *P{nos-Cas9^D10A^}attp2* or *P{nos-Cas9}attp2* embryos by microinjection. We then evaluated the heritable mutation rates as described above and found that single sgRNA never generated heritable mutants in Cas9^D10A^ transgenic flies at a rate of more than 0.1%, despite the fact that they efficiently produced mutations with an average rate of 53.1% in wild-type Cas9 nuclease flies (Table 1). To test the H840A mutation that deactivates the HNH domain, we also injected sgRNA plasmids into *P{nos-Cas9^H840A^}attp2* embryos. As expected, we did not find mutation with Cas9^H840A^ flies using one sgRNA (Table 2).

**Figure 2:**
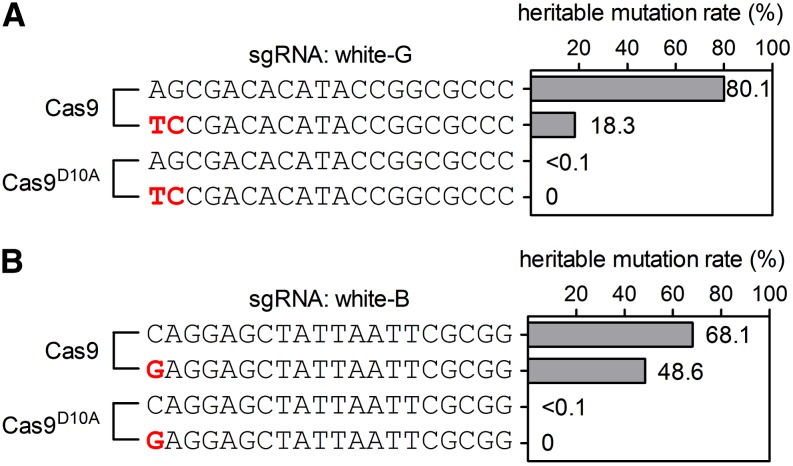
Mismatched sgRNA generates heritable mutants with Cas9 nuclease, but not with Cas9^D10A^ nickase. (A and B) Mutagenesis efficiency of sgRNAs and sgRNAs with mismatches (red). The mutagenesis efficiency of sgRNAs is tested in Cas9 or Cas9^D10A^ transgenic flies. Each row in (A) and (B) represents an sgRNA sequence and its mutagenesis rate. (A) Mutagenesis efficiency of white-G and a white-G derivative with two mismatches. (B) Mutagenesis efficiency of white-B and a white-B derivative with one mismatch. Very low mutagenesis efficiency (<0.1%) is detected with white-G and white-B when introduced into Cas9^D10A^. Mismatched sgRNAs can introduce mutations when expressed in Cas9 flies, but not with Cas9^D10A^ nickase flies.

We also tested the mutagenesis efficiency of sgRNAs with mismatches to the intended targets. We first showed that two sgRNAs generated heritable mutants at rates of 80.1% and 68.1% with the Cas9 nuclease, respectively, but almost did not generate DSBs with the Cas9 nickase (Figure 2). We then constructed two sgRNAs with one or two mismatches to the intended target and tested their mutagenesis efficiency. The mismatched sgRNA can still generate mutants at rates of 18.3% and 48.6% with the Cas9 nuclease, but not with the nickase. Although these results were not direct evaluations of off-target mutations, they suggested that one or two mismatch off-target cuts were likely to happen with wild-type Cas9, but not with Cas9 nickase.

We further examined five potential off-target sites (Figure S4) from three groups of F1 white-eyed mutants that generated from Cas9 nickase and pairs of sgRNAs. With eight genomes sequenced at each site, we did not detect any mutations induced by mismatched targeting of fewer than four nucleotides. Taken together, our *in vivo* experiments demonstrate that the application of Cas9 nickase almost avoids DSBs when guided by one sgRNA, and thus can remarkably limit off-target mutations.

### Generate *piwi* null mutant through homology-directed repair

**Figure 3:**
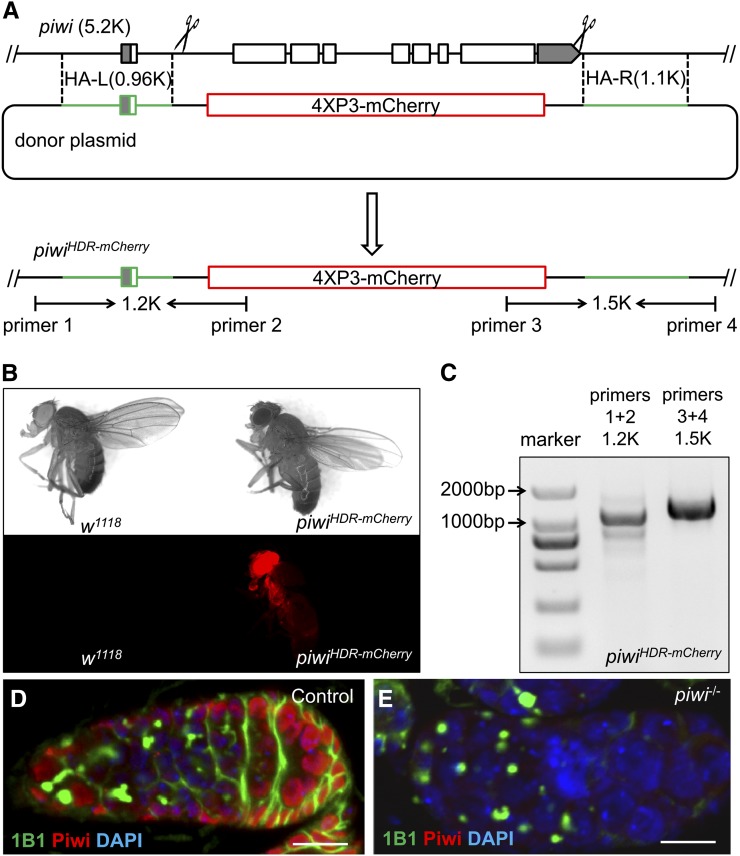
Mutagenesis through Cas9/sgRNA-induced HDR. (A) Diagrams showing the genomic region around the *piwi* locus, the donor plasmid as the repair template, and the mutation after HDR. The *piwi* donor piwi-4XP3-mCherry contains a 4XP3-mCherry sequence (red box) to replace most of the coding sequence of *piwi*. The two homologous arms (HA-L and HA-R; green) of the donor template are 0.96k bp and 1.1k bp, respectively. The *piwi* coding sequences are denoted by the white boxes, and the 5′ and 3′ UTRs are denoted by the shaded boxes. The Cas9/sgRNA cutting sites are denoted by the scissors. Successful replacement can be detected by mCherry expression in the fly eyes, or by PCR using the primer pairs shown as the arrows. (B) Images of *w^1118^* control and *piwi^HDR-mCherry^* mutant flies under bright-field (top) or epifluorescent light sources (bottom). (C) Agarose gel electrophoresis result confirming successful *piwi* HDR mutation by PCR using primers shown in (A). (D and E) Confocal images of germaria from *w^1118^* control (D) and *piwi^HDR-mCherry^* homozygous (*piwi^−/−^*; E) flies, stained with anti-Piwi (red), 1B1 (anti-Hts; green), and DAPI (blue). 1B1 shows the expression of Hu-li tai shao (Hts), a spectrosome/fusome protein. Note the morphology of the germarium is disrupted by extra GSC-like cells with round spectrosomes (E). Scale bars, 10 μm. Anterior, left.

Because Cas9^D10A^ can efficiently generate indel mutants through the NHEJ pathway, we then evaluated the efficiency of Cas9^D10A^ in HDR. Wild-type Cas9 nuclease had been successfully applied in generating mutants through HDR (Baena-Lopez *et al.* 2013; Gratz *et al.* 2014; Yu *et al.* 2014; Xue *et al.* 2014). Ideally, complete loss-of-function mutants need to dispose of entire coding sequences. To precisely replace the entire coding sequence, the approach we developed involved the transgenic Cas9 flies, two sgRNAs, and a plasmid DNA donor with a selective marker (*Materials and Methods*; Figure 3A).

We chose *piwi* for this test because known *piwi* loss-of-function mutants are homozygous viable but sterile, allowing for evaluation of the HDR efficiency of germline-essential genes. *piwi*, first discovered in *Drosophila*, is a key regulator of the expression of a group of small RNAs called *piwi*-associated RNAs (piRNAs) (Lin and Spradling 1997; Aravin *et al.* 2006; Girard *et al.* 2006; Grivna *et al.* 2006; Lau *et al.* 2006). Additionally, *piwi* is required both nonautonomously for the proper production of female germ line cells and autonomously in the germline stem cells (GSCs) before adulthood (Cox *et al.* 1998; Szakmary *et al.* 2005; Aravin *et al.* 2006; Megosh *et al.* 2006; Malone *et al.* 2009; Jin *et al.* 2013; Ma *et al.* 2014), but less is known regarding its roles in the GSCs during adulthood. Furthermore, existing *piwi* mutant alleles are *P*-element insertions or imprecise excisions of these inserts.

Through co-injection of two sgRNA plasmids targeting *piwi* and a DNA donor template with homologous arms to *piwi*, we evaluated HDR mutation rates on the *piwi* locus using embryos from *P{nos-Cas9}attp2* and *P{nos-Cas9^D10A^}attp2* flies, respectively. Successful HDR mutants were screened in the F1 generation for 4XP3 promoter-driven mCherry expression in the eyes (Figure 3B) and confirmed by PCR (Figure 3C). Wild-type Cas9 nuclease-generated heritable mutants occurred at a rate of 30.5% (111/364), whereas Cas9^D10A^ nickase yielded mutations at a very low rate (<0.3%) (Table 3).

**Table 3:** Results of mutagenesis through HDR

Method	Embryos	G0 Adult				HDR Rate, % (n)	
		Total	Survival Rate, %*	Fertile	Fertile Rate, %**	HDR-Yielding G0/Fertile G0	HDR-Positive G1/Total G1
Cas9	50	7	14	3	42.9	66.7 (2/3)	30.5 (111/364)
Cas9^D10A^	54	8	14.8	8	100	12.5 (1/8)	0.29 (3/1021)

* The survival rate is calculated as the percentage of total G0 adults divided by embryos injected.

** The fertile rate is calculated as the percentage of fertile G0 flies divided by total G0 adults.

We collected six successful F1 *piwi* HDR mutants with mCherry expression in the eyes and sequenced the break points. Three lines were generated with wild-type Cas9 and three were with Cas9^D10A^ nickase (from the same G0). All six lines had the same sequencing results at both break points (Figure S5). We then sequenced potential off-target sites of both sgRNAs in the genome (Figure S6) and found no off-target mutation in any of the six mutant lines. All six lines were homozygous viable but sterile. These mutant lines were collectively named *piwi^HDR-mCherry^*, and one line generated with Cas9^D10A^ nickase was used for the subsequent imaging experiments. Immunostaining with an antibody specifically against Piwi showed no expression of *piwi* in *piwi^HDR-mCherry^* homozygous (*piwi^−/−^*) fly ovaries, further supporting that *piwi^HDR-mCherry^* was a protein null allele (Figure 3, D and E). A previously reported germline tumor phenotype (Jin *et al.* 2013; Ma *et al.* 2014) was also observed in *piwi^−/−^* ovaries (Figure 3E).

## Discussion

Consistent with previous *in vitro* data, Cas9 nickase can efficiently generate indel mutations *in vivo* in *D. melanogaster* with a pair of sgRNAs. While this manuscript was under review, an article showing one example of using the Cas9^D10A^ nickase and one pair of sgRNAs to generate indel mutants in *yellow* was published (Port *et al.* 2014). We determined that the distance between the two sgRNA targets and their orientations are the keys to successful generation of indel mutants using the Cas9 nickase. Cas9^D10A^ is more efficient than Cas9^H840A^. The reason for this difference in performance is unclear, but recent studies of the crystal structure of Cas9 suggest that it might be related to the positioning of the incoming DNA with the Cas9-sgRNA ribonucleoprotein (Jinek *et al.* 2014; Nishimasu *et al.* 2014). In all five kinds of indel mutations generated by Cas9^D10A^ coupled with the two 11-bp-apart sgRNAs, we noticed that at least one of the sgRNA targets remains intact, which supports the notion that DSBs triggered by two sgRNAs might have occurred multiple times until one of the two targeted DNA sequences is mutated and thus is no longer recognized by the sgRNA (Cho *et al.* 2014).

Our results show that only paired sgRNAs that are close enough can generate indel mutations with Cas9 nickase. The numbers of hydrogen bonds of the base pairs located between the two nicks generated by the system might be important in determining whether DSBs happen. The epigenetic status of the genomic locus may also play a role as far-separated sgRNAs can generate mutations with Cas9 nickase in cell cultures (Cho *et al.* 2014). The orientation of the sgRNAs determines the binding orientation of Cas9 on the target DNA. Thus, the fact that only sgRNA pairs that supposedly leave a 5′ overhang can generate DSBs may reflect a physical encumbrance between the two nickases in a certain orientation when they are close on the target DNA (Nishimasu *et al.* 2014).

There are very low levels (<0.1%) of mutation when one sgRNA is introduced into Cas9^D10A^ flies. The exact mechanism of these mutations is not known, but random events such as nearby additional nicking by the base excision repair pathway might trigger DSBs. In addition, very low rates of mutation induced by DNA nicks have been reported (McConnell *et al.* 2009; Van Nierop *et al.* 2009; Certo *et al.* 2011; Chan *et al.* 2011; Davis and Maizels 2011; Metzger *et al.* 2011; Kim *et al.* 2012; Ramirez *et al.* 2012; Gabsalilow *et al.* 2013; Xu and Gupta 2013; Katz *et al.* 2014). In practice, the <0.1% mutation rate by Cas9 nickase along with one sgRNA can be ignored when inducing DSBs. In addition, we did not detect any mutations when sgRNAs carrying one or two mismatches to the DNA target were introduced into Cas9^D10A^ flies (Figure 2). These results demonstrate that the use of transgenic Cas9 nickase flies can largely avoid off-target DSBs.

Previous studies show that Cas9 nuclease is efficient in triggering HDR at a specific genomic site *in vitro* (Cong *et al.* 2013; Hsu *et al.* 2013; Ran *et al.* 2013). However, screening for null mutants is laborious and expensive. Here, we demonstrate that the donor vector carrying a selectable marker makes the procedure faster and cheaper. In contrast to wild-type Cas9 nuclease, Cas9^D10A^ nickase is not as effective in our experimental settings in generating *piwi* null mutants. One possibility is that our donor vector introduces large replacement DNA sequence into the fly genome (4.8k bp of genomic sequence with 1.9k bp of repair template) and, thus, single-strand DNA breaks may not allow the whole replacement sequence to be used as a repair template through HDR. We also cannot exclude the possibility that a NHEJ-mediated defined deletion event happens initially with the Cas9 nuclease and the two sgRNAs before HDR. This would bring the genomic regions corresponding to the two homologous arms of the donor closer for the following HDR event, which is unlikely to take place with Cas9 nickase, based on our data (Figure 1B). Finally, the orientations of the DNA nicks might affect the efficiency of HDR. Our results demonstrate that certain strategies using Cas9 nuclease in HDR may not be suitable for Cas9 nickase, but do not rule out the possibility of using Cas9 nickase in knocking-in small DNA fragments into the fly genome at a specific site through HDR.

The application of Cas9 nickase almost avoids off-target effects, and Cas9 nickases with paired offset sgRNAs can generate heritable mutants in *D. melanogaster*. These results show the advantages of applying transgenic Cas9 nickase flies, especially when off-target effects are particularly concerned. However, the mutagenesis efficiency of an sgRNA pair with the Cas9 nickase is at least two-fold lower compared with either one of the sgRNA pair using the wild-type Cas9 (Table 1). Finding and constructing a pair of sgRNAs require additional effort and time. In summary, pros and cons of the Cas9 nickase system should be weighed before deciding whether to apply the nickase or to rely on the wild-type Cas9 nuclease.

## Supplementary Material

Supporting Information
