# Supplementary material for: Performance of the Cas9 Nickase System in Drosophila melanogaster
Source: G3 (Bethesda). 2014 Aug 15;4(10):1955–62. doi: 10.1534/g3.114.013821 (PMC4199701; doi:10.1534/g3.114.013821)
Supplement: Supporting Information [file supp_4_10_1955__index.html]

Performance of the Cas9 Nickase System in Drosophila melanogaster — Supporting Information 

# Performance of the Cas9 Nickase System in *Drosophila melanogaster*

## Supporting Information for Ren *et al.*, 2014

**Files in this Data Supplement:**

- Supporting Information - Figures S1-S6 and Tables S1-S3 (PDF, 2.7 MB)
- Table S3 - Sequences of the oligonucleotides for off-target effect analysis (PDF, 74 KB)
- Figure S1 - Models of Cas9 nuclease or Cas9H840A nickase in introducing DSBs in DNA (PDF, 1.4 MB)
- Figure S2 - Full-length sequence of the nos-Cas9D10A (or *nos*-Cas9H840A) plasmid(PDF, 97 KB)
- Figure S3 - No significant improvement of survival and fertile G0 rates when applying Cas9 nickase (PDF, 720 KB)
- Figure S4 - No off-target effect detected using the transgenic Cas9 nickase flies to generate heritable mutations (PDF, 185 KB)
- Figure S5 - Representative sequencing results at the break points of the *piwiHDR-mCherry* mutant lines (PDF, 432 KB)
- Figure S6 - No off-target effect detected using the CRISPR system to generate *piwi* mutations (PDF, 206 KB)
- Table S1 - Sequences of the oligonucleotides for mutation detection and vector construction in this study (PDF, 61 KB)
- Table S2 - Sequences of the oligonucleotides for off-target effect analysis (PDF, 62 KB)
