# Supplementary material for: Performance of the Cas9 Nickase System in Drosophila melanogaster
Source: G3 (Bethesda). 2014 Aug 15;4(10):1955–62. doi: 10.1534/g3.114.013821 (PMC4199701; doi:10.1534/g3.114.013821)
Supplement: Supporting Information [file supp_g3.114.013821_013821SI.pdf]

G3 Genes | Genomes | Genetics

Performance of the Cas9 nickase system in *Drosophila melanogaster*

Xingjie Ren[*,1], Zhihao Yang[*,1], Decai Mao[*,1], Zai Chang[§,1], Huan-Huan Qiao[*,1], Xia Wang[*,1],Jin Sun[*], Qun Hu[†], Yan Cui[*], Lu-Ping Liu[*,†], Jun-Yuan Ji[‡], Jiang Xu[*,**,§§,2], and Jian-Quan Ni[*,2]

[*]Gene Regulatory Lab, School of Medicine, [§]School of Life Sciences, and [†]Tsinghua Fly Center, Tsinghua University, Beijing 100084, China; [‡]Department of Molecular and Cellular Medicine, College of Medicine, Texas A&M Health Science Center, College Station, Texas 77843, USA; [**]School of Basic Medical Sciences, Wuhan University, Wuhan 430071, China; [§§]College of Bioengineering, Hubei University of Technology, Wuhan 430068, China

[1]These authors contributed equally to this work

[2]Corresponding authors

DOI: 10.1534/g3.114.013821

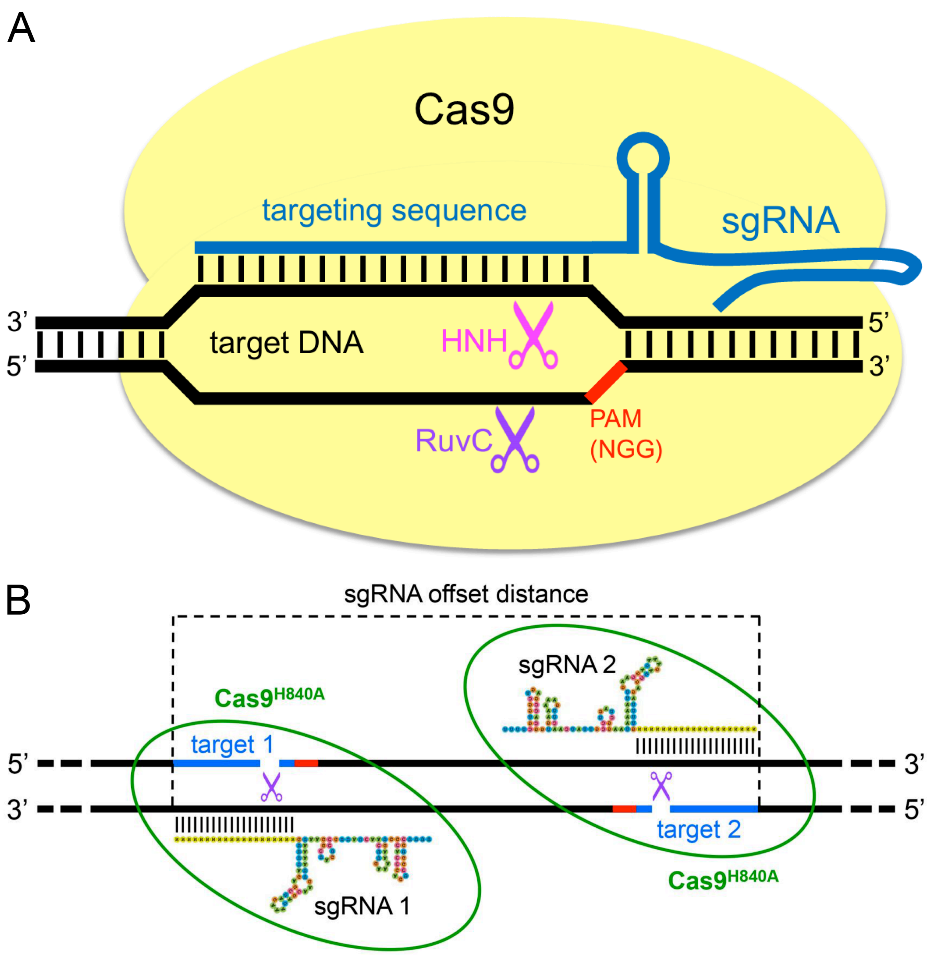

Figure S1. Models of Cas9 nuclease or Cas9^{H840A} nickase in introducing DSBs in DNA. (A) Schematic of the Cas9/sgRNA system in introducing DNA DSBs. Cas9 is shown in yellow and the chimeric sgRNA is shown in blue. The Cas9/sgRNA ribonucleoprotein recognizes the target double-stranded DNA by a 20nt complementary targeting sequence. A three nucleotide PAM sequence (NGG) is also required in the DNA for Cas9/sgRNA targeting, and is shown in red. Cas9 then introduces DSBs with the two nuclease domains. The HNH domain cuts the complementary strand to the sgRNA (magenta scissors), and the RuvC domain cuts the non-complementary strand (purple scissors). (B) Diagram showing two neighboring Cas9^{H840A}/sgRNA ribonucleoproteins targeting the fly genome. The H840A mutation converts Cas9 into a nickase that targets only the strand that is not complementary to the sgRNA. Each Cas9^{H840A} nickase is shown as a green circle. The sgRNA targets are shown in blue and the PAMs in red. The single-strand nicks are shown by the purple scissors. The offset distance is measured from the PAM-distal end of an sgRNA target to that of the other. If the PAMs are facing inward towards each other as shown in this diagram, the distance is a negative number.

GCTGCAAGGCGATTAAGTTGGGTAACGCCAGGGTTTTCCCAGTCACGACGTTGTAAAACGACGGCCAGTGAATTGTAATACGA
CTCACTATAGGGCGAATTGGGTACAAGCTTATTTATTTTGTTATGTTATATGTATTATATGTCAGACATAAAGAAAAGGAACA
CATCAAATGTGATAACAAAGACTAAACAAGTAATTTTATTACACCAAAACGACAAAACAGTAGGCAGAACAAACAACGCATAG
CCAAACATTGACGAATTGGATACCCTGCCGATTGTCAGACACTTTTGTTGATCAGTTTCTTGCGAATGGTCTCGTCCAGCGGT
GGAATCGCCTCGCGGGGAATCAGAAAAGTGGACAGATTGAACAGATCCAGAAACACCTTGTACCGATCACTGAAACCAAAAAA
AAACAAAGGGAGAACAGTTTGAGTTCATTGATCCCCGATATAATCACATCTGCGATGATCACCTGAGAGTGGAGCGCAGATAT
TGATAACCAGACGAGCCACCAGTGCCCAACTGTTGCGATCCAATCATGCGTTGCACCATGATCACGTGATTGTCTGCGGCGGG
AATAGAAAGTATTTGGTTAGGAAAACCAGTCTTAAACATAAGATATATTTATAAAAGAGTATCAAAGAATGCAATACTTACAT
CTCCACTTGGTTATTAACGAGTCGATGTCCATGAGCAGGGTGAGCAACTGGTGTGGTTGGCTGAACCTGGGTTCATCCCTATA
GAAGGTGATCATGATGGCTCCCTGAAGGGCACGATGGCTAAACCGGCGATCCCCACGACGCACCAGTGCATCGTGCACTGCCG
GATCAAAGATGGAGCGATACACCTCGCGTCGCTTCTCAATGTCCATGAGGCGGTAGTTTTTCGCCTTCTCCACGGGCTCCTCC
ATGGCGCTCTGTACCTGCGCCTCCAGGAATCGATCGACGCTCTCCTGAAACTTGGCCCAGAAGTTGAAGCCACTCTCCTCCAG
TCCGGGCGTCCTCTCCAGCCATCGCTGCACTAGCTCCAGTAGCGAGGGATCTTTCTCCGAGTTGCGAATCGAGTTCCGCGCCT
CCTCGTCGCTAAAGACATCCGAGTACTTCTGGTTGTATCTCACCCGCTGCTCTGTCAGAACTCCCAGCTTGTTCTCGATCAAA
CGGAACTGCAGCGACTGAAAACCAGATGCGGGTGCCAGGTACTTGCGGAAGTCCATGAAGTCTAGCGGGGTCATGGTCTCCAG
AATGGGCACTTGGTCCACCAGGAGCTGTACAAAGGAAGTTATAAACGGATTTTGGTAAGAGATTCAGAAAGCACTCACTTTTA
GAATCAGAACCACTCGGTTCAGTCGCTTGACAATCTCCAGCGTCTTGGTTTCATCGATGACCTCTGCATCCAACATGTCTCGT
ATGGAGTCGAACTCAAAGATGATCTGCTTGAACCAAAGCTCGTAGGCTGTGGCGAAGGTACTTAAATGCCATTGAGTGTTGTC
ATCAAAGTTGTAAACCTACTCACCCTGGTGCGTGATGATGAACAGATGCTCATCGTGCACGGGTCGCTTGTCCTCCTCGGACA
GCATACACTGGGCATCCAGCAGTTTGTCCAGCATCAGATACTCTCCATAGATTTTGCCCACTTCCGTGGTTAATGGCACCGCC
GAATCATCGTGATCGTTTCTGTATGGGTTTGAATTGAATCGCAGAACTGAAGATCGATTGGCATTCCTGGACAGCACGTGCTG
GTGCTCACCCGTTTCCTGCATAGGGACAGCTCATGGTGCACAGCTCAGATCAGATCGTGACTCCTCGACCGGCGGATGCTGGC
GAACTGATCTCCGCCAGCGGACCGGAGATGAGACCCCAGCGAACCGATAACAGAGCGAGAGAGCTCCAGTTCCGACTGATTGC
ACAGTCGGTGATCTGGGCGATGGGCACTGCCAGATAGGCTGGGAATTATCAATCACTTGAGGTGAAAGTGCGGCGCACACAAA
TCCAAGCTTGATATCATCGATCTCGACGCTGCATCCAACGCGTTGGGAGCTCTCCGGATCAATTCGGCTTCACGTACCGTCGA
CGATGTAGGTCACGGTCTCGAAGCCGCGGTGCGGGTGCCAGGGCGTGCCCTTGGGCTCCCCGGGCGCGTACTCCACCTCACCC
ATCTGGTCCATCATGATGAACGGGTCGAGGTGGCGGTAGTTGATCCCGGCGAACGCGCGGCGCACCGGGAAGCCCTCGCCCTC
GAAACCGCTGGGCGCGGTGGTCACGGTGAGCACGGGACGTGCGACGGCGTCGGCTGGTGCGGATACGCGGGGCAGCGTCAGCG
GGTTCTCGACGGTCACGGCGGGCATGTCGACAAGCCGAATTGATCCACTAGAAGGCCTAATTCGtCTAGCAGGATCTctaggG
CTTCGACCGTTTTAACCTCGAAATATGCACATGTAAGGACGGATGTGAGCGAACGCCAGTGATGACCGGGATCAGAGGTAACC
TACCATGGTGGGGATTAGGTGACCGTTCGCAGGTAGTTTGATCGGAGCGAATGTTCGGGGGGTCTGGCGTCAGAGGCTCTAAA
CTTTATGTAATTCCTGCCGCGAAACACGCACGTATCAAGCAGTCAGCTGTTCTCTTCGTTCAGCGCGCGCCGGTGTTGCAAAA
CGAGCGCTCTTCGCCGGCGGTGGCTCGTGCGATAGTTCGTTTTGTCGGTAATCCGATGTTGCCGCGCCGATATCATGTGATGT
TGTCACAGTGCGCGAAATTCGAATGGTGGTGTGCAGTGATTGTGTTGTGACGGCGAGTGGCGCGTGTGGGTGCTTAGTTTTGG
GAGATGTTTTCGTATTTTTTTGTTGATAACTCAGGCTTTGTTGCTGTGTTGTAGTACTATTTTCCATTGCGCGGTGTCCAGCT
TTTAATTAGTGGCACATATTCTTAGCAAGTAAAAATTATTTTGCATACTATTAAATTTCTTATAAATTATTTTCTAAAATTAA
GTTTACCTTTTCAATTTTACTAAAAATATCGATATATTTATTATCGCTGGAAAACTACATTATTCCACCTCTAAGCAAGAACC
GTTAGTTGGCGCGTAGCTTTACCACAAAATTCCTGGAATTGCCGTACGCTTCGCAGTTGTTTCAAGTTGTCTAAGGGACATAC
GATTTTTTTTGCCTCTGCGTCACGATTTTAACCCAAAAGCGAGTTTAGTTACATGTACATTATTATTAGATAAAGAAGTATCG
CGAATACTTCAGTTGAATAAACTGTGCTTGGTTTTTGGGTGAGGATTTGTGGAAAGTAGAGTGCGCGATAACCGTAACTTTCG
ACCCGGATTTTCGCCtgAATTgagatctcTCTAGAggtacCGCCACCATGGCCCCAAAGAAGAAGCGGAAGGTCGGTATCCAC
GGTGTCCCAGCAGCCATGGACAAGAAGTACTCCATTGGGCTC**GCT**ATCGGCACAAACAGCGTCGGCTGGGCCGTCATTACGGA
CGAGTACAAGGTGCCGAGCAAAAAATTCAAAGTTCTGGGCAATACCGATCGCCACAGCATAAAGAAGAACCTCATTGGCGCCC
TCCTGTTCGACTCCGGGGAGACGGCCGAAGCCACGCGGCTCAAAAGAACAGCACGGCGCAGATATACCCGCAGAAAGAATCGG
ATCTGCTACCTGCAGGAGATCTTTAGTAATGAGATGGCTAAGGTGGATGACTCTTTCTTCCATAGGCTGGAGGAGTCCTTTTT
GGTGGAGGAGGATAAAAAGCACGAGCGCCACCCAATCTTTGGCAATATCGTGGACGAGGTGGCGTACCATGAAAAGTACCCAA
CCATATATCATCTGAGGAAGAAGCTTGTAGACAGTACTGATAAGGCTGACTTGCGGTTGATCTATCTCGCGCTGGCGCATATG
ATCAAATTTCGGGGACACTTCCTCATCGAGGGGGACCTGAACCCAGACAACAGCGATGTCGACAAACTCTTTATCCAACTGGT
TCAGACTTACAATCAGCTTTTCGAAGAGAACCCGATCAACGCATCCGGAGTTGACGCCAAAGCAATCCTGAGCGCTAGGCTGT
CCAAATCCCGGCGGCTCGAAAACCTCATCGCACAGCTCCCTGGGGAGAAGAAGAACGGCCTGTTTGGTAATCTTATCGCCCTG
TCACTCGGGCTGACCCCCAACTTTAAATCTAACTTCGACCTGGCCGAAGATGCCAAGCTTCAACTGAGCAAAGACACCTACGA
TGATGATCTCGACAATCTGCTGGCCCAGATCGGCGACCAGTACGCAGACCTTTTTTTGGCGGCAAAGAACCTGTCAGACGCCA
TTCTGCTGAGTGATATTCTGCGAGTGAACACGGAGATCACCAAAGCTCCGCTGAGCGCTAGTATGATCAAGCGCTATGATGAG
CACCACCAAGACTTGACTTTGCTGAAGGCCCTTGTCAGACAGCAACTGCCTGAGAAGTACAAGGAAATTTTCTTCGATCAGTC
TAAAAATGGCTACGCCGGATACATTGACGGCGGAGCAAGCCAGGAGGAATTTTACAAATTTATTAAGCCCATCTTGGAAAAAA
TGGACGGCACCGAGGAGCTGCTGGTAAAGCTTAACAGAGAAGATCTGTTGCGCAAACAGCGCACTTTCGACAATGGAAGCATC
CCCCACCAGATTCACCTGGGCGAACTGCACGCTATcCTCAGGCGGCAAGAGGATTTCTACCCCTTTTTGAAAGATAACAGGGA
AAAGATTGAGAAAATCCTCACATTTCGGATACCCTACTATGTAGGCCCCCTCGCCCGGGGAAATTCCAGATTCGCGTGGATGA

CTCGCAAATCAGAAGAGACCATCACTCCCTGGAACTTCGAGGAAGTCGTGGATAAGGGGGCCTCTGCCCAGTCCTTCATCGAA
AGGATGACTAACTTTGATAAAAATCTGCCTAACGAAAAGGTGCTTCCTAAACACTCTCTGCTGTACGAGTACTTCACAGTTTA
TAACGAGCTCACCAAGGTCAAATACGTCACAGAAGGGATGAGAAAGCCAGCATTCCTGTCTGGAGAGCAGAAGAAAGCTATCG
TGGACCTCCTCTTCAAGACGAACCGGAAAGTTACCGTGAAACAGCTCAAAGAAGACTATTTCAAAAAGATTGAATGTTTCGAC
TCTGTTGAAATCAGCGGAGTGGAGGATCGCTTCAACGCATCCCTGGGAACGTATCACGATCTCCTGAAAATCATTAAAGACAA
GGACTTCCTGGACAATGAGGAGAACGAGGACATTCTTGAGGACATTGTCCTCACCCTTACGTTGTTTGAAGATAGGGAGATGA
TTGAAGAACGCTTGAAAACTTACGCTCATCTCTTCGACGACAAAGTCATGAAACAGCTCAAGAGGCGCCGATATACAGGATGG
GGGCGGCTGTCAAGAAAACTGATCAATGGGATCCGAGACAAGCAGAGTGGAAAGACAATCCTGGATTTTCTTAAGTCCGATGG
ATTTGCCAACCGGAACTTCATGCAGTTGATCCATGATGACTCTCTCACCTTTAAGGAGGACATCCAGAAAGCACAAGTTTCTG
GCCAGGGGGACAGTCTTCACGAGCACATCGCTAATCTTGCAGGTAGCCCAGCTATCAAAAAGGGAATACTGCAGACCGTTAAG
GTCGTGGATGAACTCGTCAAAGTAATGGGAAGGCATAAGCCCGAGAATATCGTTATCGAGATGGCCCGAGAGAACCAAACTAC
CCAGAAGGGACAGAAGAACAGTAGGGAAAGGATGAAGAGGATTGAAGAGGGTATAAAAGAACTGGGGTCCCAAATCCTTAAGG
AACACCCAGTTGAAAACACCCAGCTTCAGAATGAGAAGCTCTACCTGTACTACCTGCAGAACGGCAGGGACATGTACGTGGAT
CAGGAACTGGACATCAATCGGCTCTCCGACTACGACGTGGAT**GCT**ATCGTGCCCCAGTCTTTTCTCAAAGATGATTCTATTGA
TAATAAAGTGTTGACAAGATCCGATAAAAATAGAGGGAAGAGTGATAACGTCCCCTCAGAAGAAGTTGTCAAGAAAATGAAAA
ATTATTGGCGGCAGCTGCTGAACGCCAAACTGATCACACAACGGAAGTTCGATAATCTGACTAAGGCTGAACGAGGTGGCCTG
TCTGAGTTGGATAAAGCcGGCTTCATCAAAAGGCAGCTTGTTGAGACACGCCAGATCACCAAGCACGTGGCCCAAATTCTCGA
TTCACGCATGAACACCAAGTACGATGAAAATGACAAACTGATTCGAGAGGTGAAAGTTATTACTCTGAAGTCTAAGCTGGTCT
CAGATTTCAGAAAGGACTTTCAGTTTTATAAGGTGAGAGAGATCAACAATTACCACCATGCGCATGATGCCTACCTGAATGCA
GTGGTAGGCACTGCACTTATCAAAAAATATCCCAAGCTTGAATCTGAATTTGTTTACGGAGACTATAAAGTGTACGATGTTAG
GAAAATGATCGCAAAGTCTGAGCAGGAAATAGGCAAGGCCACCGCTAAGTACTTCTTTTACAGCAATATTATGAATTTTTTCA
AGACCGAGATTACACTGGCCAATGGAGAGATTCGGAAGCGACCACTTATCGAAACAAACGGAGAAACAGGAGAAATCGTGTGG
GACAAGGGTAGGGATTTCGCGACAGTCCGGAAGGTCCTGTCCATGCCGCAGGTGAACATCGTTAAAAAGACCGAAGTACAGAC
CGGAGGCTTCTCCAAGGAAAGTATCCTCCCGAAAAGGAACAGCGACAAGCTGATCGCACGCAAAAAAGATTGGGACCCCAAGA
AATACGGCGGATTCGATTCTCCTACAGTCGCTTACAGTGTACTGGTTGTGGCCAAAGTGGAGAAAGGGAAGTCTAAAAAACTC
AAAAGCGTCAAGGAACTGCTGGGCATCACAATCATGGAGCGATCAAGCTTCGAAAAAAACCCCATCGACTTTCTCGAGGCGAA
AGGATATAAAGAGGTCAAAAAAGACCTCATCATTAAGCTTCCCAAGTACTCTCTCTTTGAGCTTGAAAACGGCCGGAAACGAA
TGCTCGCTAGTGCGGGCGAGCTGCAGAAAGGTAACGAGCTGGCACTGCCCTCTAAATACGTTAATTTCTTGTATCTGGCCAGC
CACTATGAAAAGCTCAAAGGGTCTCCCGAAGATAATGAGCAGAAGCAGCTGTTCGTGGAACAACACAAACACTACCTTGATGA
GATCATCGAGCAAATAAGCGAATTCTCCAAAAGAGTGATCCTCGCCGACGCTAACCTCGATAAGGTGCTTTCTGCTTACAATA
AGCACAGGGATAAGCCCATCAGGGAGCAGGCAGAAAACATTATCCACTTGTTTACTCTGACCAACTTGGGCGCGCCTGCAGCC
TTCAAGTACTTCGACACCACCATAGACAGAAAGCGGTACACCTCTACAAAGGAGGTCCTGGACGCCACACTGATTCATCAGTC
AATTACGGGGCTCTATGAAACAAGAATCGACCTCTCTCAGCTCGGTGGAGACAAGCGTCCTGCTGCTACTAAGAAAGCTGGTC
AAGCTAAGAAAAAGAAATAATctagcacctaggaggatccactcgAGGTTTAGAGAGGGCGAATCCAGCTCTGGAGCAGAGGC
TCTGGCAGCTTTTGCAGCGTTTATATAACATGAAATATATATACGCATTCCGATCAAAGCTGGGTTAACCAGATAGATAGATA
GTAACGTTTAAATAGCGCCTGGCGCGTTCGATTTTAAAGAGATTTAGAGCGTTATCCCGTGCCTATAGATCaTATAGTATAGA
CAACGAACGATCACTCAAATCCAAGTCAATAATTCAAGAATTTATGTCTGTTTCTGTGAAAGGGAAACTAATTTTGTTAAAGA
AGACTTACAATATCGTAATACTTGTTCAATCGTCGTGGCCGATAGAAATATCTTACAATCCGAAAGTTGATGAATGGAATTGG
TCTGCAACTGGTCGCCTTCATTTCGTAAAATGTTCGCTTGCGGCCGAAAAATTTCGATATATCTACAATTcATCTACAATCTT
TACTAAATTTTGAAAAAGGAACACTTTGAATTTCGAACTGTCAATCGTATCATTAGAATTTAATCTAAATTTAAATCTTGCTA
AAGGAAATAGCAAGGAACACTTTCGTCGTCGGCTACGCATTCATTGTAAAATTTTAAATTTTGACATTCCGCACTTTTTGATA
GATAAGCGAAGAGTATTTTTATTACATGTATCGCAAGTATTCATTTCAACACACATATCTATATATATATATATATATATATA
TATATATATATATATATATGTTATATATTTATTCAATTTTGTTTACCATTGATCAATTTTTCACACATGAAACAACCGCCAGC
ATTATATAATTTTTTTATTTTTTTAAAAAATGTGTACACATATTCTGAAAATGAAAAATTCAATGGCTCGAcTGCCAAATAAA
GAAATGGTTACAATTTAAGGAAACAAATGTCCTTCTTGCACTAGTGAGCTCCAGCTTTTGTTCCCTTTAGTGAGGGTTAATTT
CGAGCTTGGCGTAATCATGGTCATAGCTGTTTCCTGTGTGAAATTGTTATCCGCTCACAATTCCACACAACATACGAGCCGGA
AGCATAAAGTGTAAAGCCTGGGGTGCCTAATGAGTGAGCTAACTCACATTAATTGCGTTGCGCTCACTGCCCGCTTTCCAGTC
GGGAAACCTGTCGTGCCAGCTGCATTAATGAATCGGCCAACGCGCGGGGAGAGGCGGTTTGCGTATTGGGCGCTCTTCCGCTT
CCTCGCTCACTGACTCGCTGCGCTCGGTCGTTCGGCTGCGGCGAGCGGTATCAGCTCACTCAAAGGCGGTAATACGGTTATCC
ACAGAATCAGGGGATAACGCAGGAAAGAACATGTGAGCAAAAGGCCAGCAAAAGGCCAGGAACCGTAAAAAGGCCGCGTTGCT
GGCGTTTTTCCATAGGCTCCGCCCCCCTGACGAGCATCACAAAAATCGACGCTCAAGTCAGAGGTGGCGAAACCCGACAGGAC
TATAAAGATACCAGGCGTTTCCCCCTGGAAGCTCCCTCGTGCGCTCTCCTGTTCCGACCCTGCCGCTTACCGGATACCTGTCC
GCCTTTCTCCCTTCGGGAAGCGTGGCGCTTTCTCATAGCTCACGCTGTAGGTATCTCAGTTCGGTGTAGGTCGTTCGCTCCAA
GCTGGGCTGTGTGCACGAACCCCCCGTTCAGCCCGACCGCTGCGCCTTATCCGGTAACTATCGTCTTGAGTCCAACCCGGTAA
GACACGACTTATCGCCACTGGCAGCAGCCACTGGTAACAGGATTAGCAGAGCGAGGTATGTAGGCGGTGCTACAGAGTTCTTG
AAGTGGTGGCCTAACTACGGCTACACTAGAAGGACAGTATTTGGTATCTGCGCTCTGCTGAAGCCAGTTACCTTCGGAAAAAG
AGTTGGTAGCTCTTGATCCGGCAAACAAACCACCGCTGGTAGCGGTGGTTTTTTTGTTTGCAAGCAGCAGATTACGCGCAGAA
AAAAAGGATCTCAAGAAGATCCTTTGATCTTTTCTACGGGGTCTGACGCTCAGTGGAACGAAAACTCACGTTAAGGGATTTTG

GTCATGAGATTATCAAAAAGGATCTTCACCTAGATCCTTTTAAATTAAAAATGAAGTTTTAAATCAATCTAAAGTATATATGA
GTAAACTTGGTCTGACAGTTACCAATGCTTAATCAGTGAGGCACCTATCTCAGCGATCTGTCTATTTCGTTCATCCATAGTTG
CCTGACTCCCCGTCGTGTAGATAACTACGATACGGGAGGGCTTACCATCTGGCCCCAGTGCTGCAATGATACCGCGAGACCCA
CGCTCACCGGCTCCAGATTTATCAGCAATAAACCAGCCAGCCGGAAGGGCCGAGCGCAGAAGTGGTCCTGCAACTTTATCCGC
CTCCATCCAGTCTATTAATTGTTGCCGGGAAGCTAGAGTAAGTAGTTCGCCAGTTAATAGTTTGCGCAACGTTGTTGCCATTG
CTACAGGCATCGTGGTGTCACGCTCGTCGTTTGGTATGGCTTCATTCAGCTCCGGTTCCCAACGATCAAGGCGAGTTACATGA
TCCCCCATGTTGTGCAAAAAAGCGGTTAGCTCCTTCGGTCCTCCGATCGTTGTCAGAAGTAAGTTGGCCGCAGTGTTATCACT
CATGGTTATGGCAGCACTGCATAATTCTCTTACTGTCATGCCATCCGTAAGATGCTTTTCTGTGACTGGTGAGTACTCAACCA
AGTCATTCTGAGAATAGTGTATGCGGCGACCGAGTTGCTCTTGCCCGGCGTCAATACGGGATAATACCGCGCCACATAGCAGA
ACTTTAAAAGTGCTCATCATTGGAAAACGTTCTTCGGGGCGAAAACTCTCAAGGATCTTACCGCTGTTGAGATCCAGTTCGAT
GTAACCCACTCGTGCACCCAACTGATCTTCAGCATCTTTTACTTTCACCAGCGTTTCTGGGTGAGCAAAAACAGGAAGGCAAA
ATGCCGCAAAAAAGGGAATAAGGGCGACACGGAAATGTTGAATACTCATACTCTTCCTTTTTCAATATTATTGAAGCATTTAT
CAGGGTTATTGTCTCATGAGCGGATACATATTTGAATGTATTTAGAAAAATAAACAAATAGGGGTTCCGCGCACATTTCCCCG
AAAAGTGCCACCTAAATTGTAAGCGTTAATATTTTGTTAAAATTCGCGTTAAATTTTTGTTAAATCAGCTCATTTTTTAACCA
ATAGGCCGAAATCGGCAAAATCCCTTATAAATCAAAAGAATAGACCGAGATAGGGTTGAGTGTTGTTCCAGTTTGGAACAAGA
GTCCACTATTAAAGAACGTGGACTCCAACGTCAAAGGGCGAAAAACCGTCTATCAGGGCGATGGCCCACTACGTGAACCATCA
CCCTAATCAAGTTTTTTGGGGTCGAGGTGCCGTAAAGCACTAAATCGGAACCCTAAAGGGAGCCCCCGATTTAGAGCTTGACG
GGGAAAGCCGGCGAACGTGGCGAGAAAGGAAGGGAAGAAAGCGAAAGGAGCGGGCGCTAGGGCGCTGGCAAGTGTAGCGGTCA
CGCTGCGCGTAACCACCACACCCGCCGCGCTTAATGCGCCGCTACAGGGCGCGTCCCATTCGCCATTCAGGCTGCGCAACTGT
TGGGAAGGGCGATCGGTGCGGGCCTCTTCGCTATTACGCCAGCTGGCGAAAGGGGGATGT

Figure S2. Full-length sequence of the *nos*-Cas9^{D10A} (or *nos*-Cas9^{H840A}) plasmid. The key components of the plasmid are color-coded. The *vermilion* reporter, magenta; *attB* site, brown; the *nos* promoter, 5'UTR, and 3'UTR, cyan; *Cas9* gene, green; the D10A mutation, red (or the H840A mutation, purple).

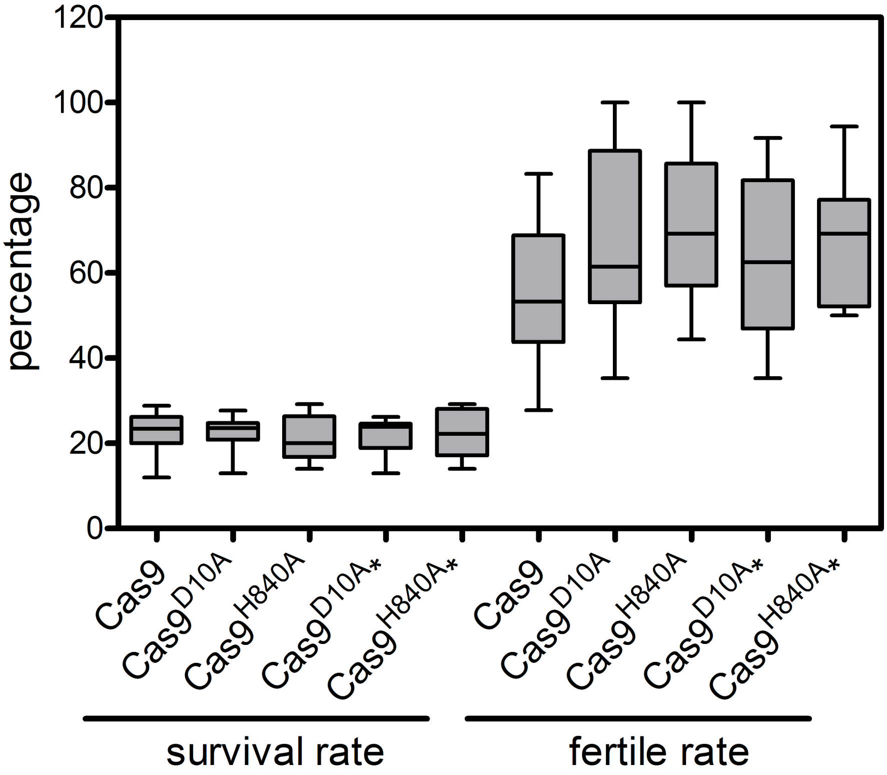

Figure S3. No significant improvement of survival and fertile G0 rates when applying Cas9 nickase. Using data from Table S3, the average survival and fertile G0 rates are calculated for Cas9 with one sgRNA (Cas9), or for Cas9 nickase with offset sgRNA pairs ($Cas9^{D10A}$ and $Cas9^{H840A}$). In addition, survival and fertile G0 rates for Cas9 nickase with paired sgRNAs that successfully generated heritable mutants are also calculated ($Cas9^{D10A*}$ and $Cas9^{H840A*}$). No significant differences are observed among five groups, for either survival rate or fertile rate.

NGG:PAM **N**:mismatch

CAATATGGACATCTTTGGGG**CGG** white-sgRNA-A

TA**T**TA**A**GGACATCTTTGGG**TCGG** potential off-target site A1

CAGGAGCTATTAATTCGCGG**AGG** white-sgRNA-B

CAGGAGCTATTAAT**GTC**CG**CCGG** potential off-target site B1

GCACAATATGGACATCTTTG**GGG** white-sgRNA-C

CC**TG**AA**C**ATGGACATCTTTG**CAG** potential off-target site C1

AGGTGAGGTTCTCGGCTAGT**TGG** white-sgRNA-D

A**AC**TGA**T**G**A**TCTCGGCTAGT**TGG** potential off-target site D1

GG**A**TGAGGTTCTCGGCT**G**G**CTGG** potential off-target site D2

Figure S4. No off-target effect detected using the transgenic Cas9 nickase flies to generate heritable mutations. F1 flies from three groups of successful *white* mutations using $Cas9^{D10A}$ flies and pairs of sgRNAs are tested. Four sgRNA on-target sequences and their corresponding potential off-target sites are listed. Genomic regions that have no more than four mismatches to the on-target and a neighboring PAM are sequenced from eight F1 white-eyed flies for each group. No mutations were detected at the potential off-targets. Mismatches between the potential off-targets and the targeted region are shown in red. The PAM sequences are in bold type.

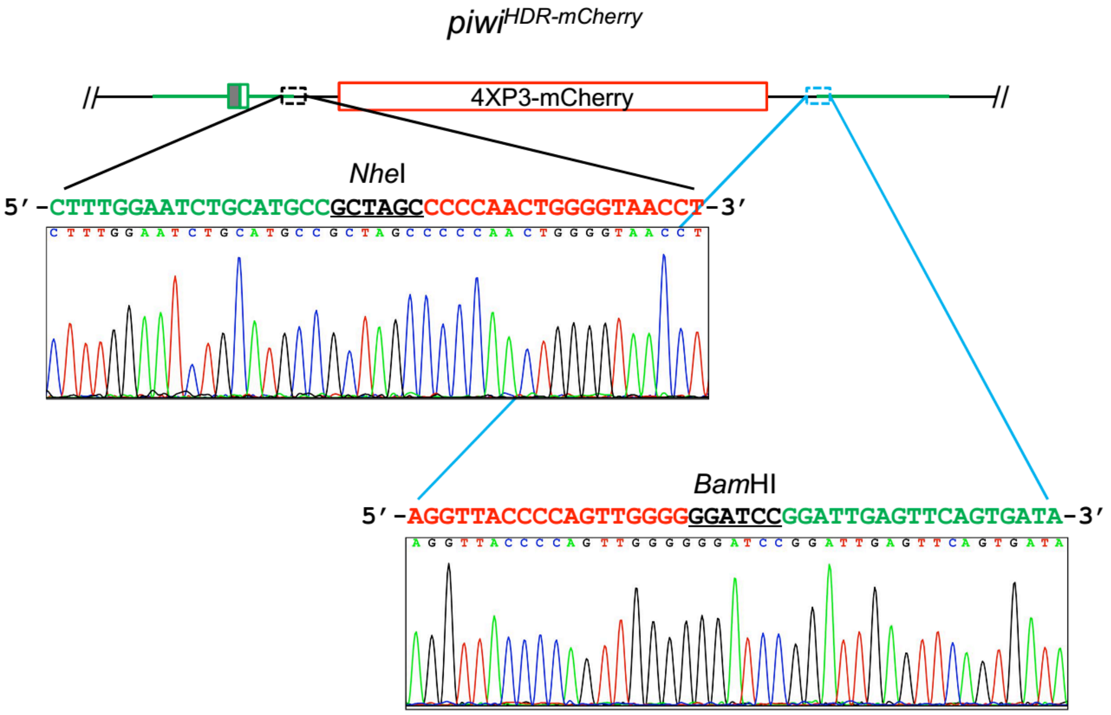

Figure S5. Representative sequencing results at the break points of the *piwi*$^{HDR\text{-}mCherry}$ mutant lines. The two boxes show parts of the sequencing results that represent the break points on the genomic DNA, with the genomic sequence in green and the 4XP3-mCherry sequence in red. Sequencing results with all six *piwi*$^{HDR\text{-}mCherry}$ lines show the same result.

NGG:PAM **N**:mismatch

piwi-HDR-sgRNA-left

CGGACATGACCAAATGGACC**CGG**

CGGACATGACCAAAT**T**GAC**T****CGG** potential off-target site L1

CGGACATGACCA**G**AT**C**GACC**GGG** potential off-target site L2

CGGACATGACCAAAT**C**GA**GT****CGG** potential off-target site L3

CGGACATGACCA**G**AT**C**GAC**T****AGG** potential off-target site L4

CGGACATGACCA**G**AT**C**GAC**T****CAG** potential off-target site L5

piwi-HDR-sgRNA-right

TATCGATTGCAAGTTGGGGT**AGG**

TATCGATTGCAA**A**TTG**T**G**CC****TGG** potential off-target site R1

TATCG**T**TTGCAAGTTG**CTA**T**CAG** potential off-target site R2

Figure S6. No off-target effect detected using the CRISPR system to generate *piwi* mutations. Flies from six successful *piwi*$^{HDR\text{-}mCherry}$ mutation strains are tested. The two sgRNA on-target sequences and their corresponding potential off-target sites are listed. Genomic regions that have no more than four mismatches to the on-target and a neighboring PAM are sequenced. No mutations were detected at the potential off-targets. Mismatches between the potential off-targets and the targeted region are shown in red. The PAM sequences are in bold type.

Table S1. Sequences of the oligonucleotides for mutation detection and vector construction in this study.

Use	Name	Sequence
Indel mutation detection	White-F	TATCATTGCAGGGTGACAGCG
	White-R	GGCATTCAGCAGGGTCGTC
Cas9 nickase mutagenesis	Cas9-D10A-F	CTCCATTGGGCTCGCTATCGGCACAAACA
	Cas9-D10A-R	TGTTTGTGCCGATAGCGAGCCCAATGGAG
	Cas9-H840A-F	TACGACGTGGATGCTATCGTGCCCCAG
	Cas9-H840A-R	CTGGGGCACGATAGCATCCACGTCGTA
piwi-4XP3-mCherry donor template homologous arms	piwi-L-F	GGCCTAGGTGCGGTATTTAAGGCTCTTGC
	piwi-L-R	CTAGCTAGCGGCATGCAGATTCCAAAGAC
	piwi-R-F	CGGGATCCGGATTGAGTTCAGTGATAATATCG
	piwi-R-R	GGACTAGTCGCAACTGAGCAATACTTTCCAC
piwi-4XP3-mCherry donor template mCherry marker	mCherry-F	CCGCTCGAGATGGTGAGCAAGGGCGAGGA
	mCherry-R	CGGGGTACCTTACTTGTACAGCTCGTCCATGCC
	SV40-F	CGGGGTACCGATCTTTGTGAAGGAACC
	SV40-R	CGGGATATCACTAGACTGGAACCAGACATG
$piwi^{HDR\text{-}mCherry}$ confirmation	primer 1	TTACTTGCATAGCAGGGTGGCTG
	primer 2	CTCCGGCGCTCGTCTAGTGTCG
	primer 3	CACACCTCCCCCTGAACCTG
	primer 4	GCCTCCACCAGTTCCAGACG

Table S2. Sequences of the oligonucleotides for off-target effect analysis.

primers	Sequence (5' to 3')
white-OTE-A1-F	CGACACCAGCTATGGTTCGG
white-OTE-A1-R	CTCGGTATGGACCTAGAGGC
white-OTE-B1-F	AACAAACAACCGCTCAACCC
white-OTE-B1-R	GAATCGGGCGTAGAAGACAG
white-OTE-C1-F	TGTTCGTCGTCCTCTTCCTCC
white-OTE-C1-R	AGGAGCACAAGGCGAAGCAC
white-OTE-D1-F	ACCAAGGGTGGATAGGTGCG
white-OTE-D1-R	GGGTTGAAACTTCCTACTTGCC
white-OTE-D2-F	GATCTTTGGCTTTCGTTCAGG
white-OTE-D2-R	TTGAACCGCCCAACTGCTCC
piwi-OTE-L1-F	GAACCTGAATGCCTAATCTC
piwi-OTE-L1-R	CGATTTGACTTTGGGCAAGA
piwi-OTE-L2-F	CAACTGACCTCTTAGCCCTCG
piwi-OTE-L2-R	TAGAGCGAGATTTCCTTGAGC
piwi-OTE-L3-F	ACTGGGACCTACGATTGCC
piwi-OTE-L3-R	AAGCGAGACCGCACTACCG
piwi-OTE-L4-F	GGAGGGTAAAGGGCACGAAA
piwi-OTE-L4-F	CAATGAGTTTGCCCTTGACG
piwi-OTE-L5-F	CGATAGAAATGGGCAAAGCAAC
piwi-OTE-L5-R	GTGGAGGTGGTATTTATTGCCC
piwi-OTE-R1-F	GTCATTAAGTCTGGCCTGTGGG
piwi-OTE-R1-R	AGGGCTCAATGTTGAACGAAGC
piwi-OTE-R2-F	CATCGCATCCATGATTTCCTC
piwi-OTE-R2-R	AGGCGCAAAGTATTTCACCC

Table S3. Sequences of the oligonucleotides for off-target effect analysis.

	sgRNA name	embryos	total GO	fertile G0	survival rate*	fertile rate**
Cas9	white-A	53	12	8	22.6%	66.7%
	white-B	61	16	13	26.2%	81.3%
	white-C	60	15	8	25.0%	53.3%
	white-D	50	10	5	20.0%	50.0%
	white-E	51	10	8	19.6%	80.0%
	white-F	65	16	7	24.6%	43.8%
	white-G	65	16	11	24.6%	68.8%
	white-H	65	14	9	21.5%	64.3%
	white-I	50	6	5	12.0%	83.3%
	white-J	50	10	5	20.0%	50.0%
	white-K	66	19	6	28.8%	31.6%
	white-L	64	15	8	23.4%	53.3%
	white-M	65	15	5	23.1%	33.3%
	white-N	66	18	12	27.3%	66.7%
	white-O	60	16	5	26.7%	31.3%
	white-P	65	18	5	27.7%	27.8%
	white-Q	60	12	9	20.0%	75.0%
	white-R	55	8	4	14.5%	50.0%
	white-S	65	16	10	24.6%	62.5%
Cas9^{D10A}	white-J + white-F	55	11	7	20.0%	63.6%
	white-E + white-K	56	13	8	23.2%	61.5%
	white-E + white-L	60	15	9	25.0%	60.0%
	white-E + white-F	65	17	10	26.2%	58.8%
	white-D + white-R	54	7	6	13.0%	85.7%
	white-D + white-H	65	18	8	27.7%	44.4%
	white-Q + white-B	56	10	7	17.9%	70.0%
	white-E + white-R	68	16	11	23.5%	68.8%
	white-D + white-I	66	16	9	24.2%	56.3%
	white-D + white-C	55	12	11	21.8%	91.7%
	white-E + white-H	65	16	7	24.6%	43.8%
	white-E + white-I	65	16	9	24.6%	56.3%
	white-E + white-A	65	17	6	26.2%	35.3%
	white-F + white-A	65	16	8	24.6%	50.0%
	white-E + white-G	50	9	9	18.0%	100.0%
	white-E + white-A	55	13	13	23.6%	100.0%
	white-E + white-O	60	13	12	21.7%	92.3%
Cas9^{H840A}	white-K + white-S	65	17	9	26.2%	52.9%
	white-F + white-L	65	19	13	29.2%	68.4%
	white-K + white-P	55	10	7	18.2%	70.0%
	white-F + white-M	55	10	5	18.2%	50.0%
	white-S + white-N	65	18	17	27.7%	94.4%
	white-E + white-F	50	7	5	14.0%	71.4%
	white-D + white-R	68	18	11	26.5%	61.1%
	white-D + white-H	50	9	4	18.0%	44.4%
	white-Q + white-B	45	7	6	15.6%	85.7%
	white-E + white-R	45	7	6	15.6%	85.7%
	white-D + white-C	55	13	9	23.6%	69.2%
	white-E + white-H	50	10	10	20.0%	100.0%
	white-E + white-I	65	16	11	24.6%	68.8%

*The survival rate is calculated as the percentage of total G0 adults divided by embryos injected.

**The fertile rate is calculated as the percentage of fertile G0 flies divided by total G0 adults.
