# Supplementary material for: Performance of the Cas9 Nickase System in Drosophila melanogaster
Source: G3 (Bethesda). 2014 Aug 15;4(10):1955–62. doi: 10.1534/g3.114.013821 (PMC4199701; doi:10.1534/g3.114.013821)
Supplement: Supporting Information [file supp_g3.114.013821_TableS3.pdf]

Table S3. Sequences of the oligonucleotides for off-target effect analysis.

	sgRNA name	embryos	total GO	fertile G0	survival rate*	fertile rate**
Cas9	white-A	53	12	8	22.6%	66.7%
	white-B	61	16	13	26.2%	81.3%
	white-C	60	15	8	25.0%	53.3%
	white-D	50	10	5	20.0%	50.0%
	white-E	51	10	8	19.6%	80.0%
	white-F	65	16	7	24.6%	43.8%
	white-G	65	16	11	24.6%	68.8%
	white-H	65	14	9	21.5%	64.3%
	white-I	50	6	5	12.0%	83.3%
	white-J	50	10	5	20.0%	50.0%
	white-K	66	19	6	28.8%	31.6%
	white-L	64	15	8	23.4%	53.3%
	white-M	65	15	5	23.1%	33.3%
	white-N	66	18	12	27.3%	66.7%
	white-O	60	16	5	26.7%	31.3%
	white-P	65	18	5	27.7%	27.8%
	white-Q	60	12	9	20.0%	75.0%
	white-R	55	8	4	14.5%	50.0%
	white-S	65	16	10	24.6%	62.5%
$Cas9^{D10A}$	white-J + white-F	55	11	7	20.0%	63.6%
	white-E + white-K	56	13	8	23.2%	61.5%
	white-E + white-L	60	15	9	25.0%	60.0%
	white-E + white-F	65	17	10	26.2%	58.8%
	white-D + white-R	54	7	6	13.0%	85.7%
	white-D + white-H	65	18	8	27.7%	44.4%
	white-Q + white-B	56	10	7	17.9%	70.0%
	white-E + white-R	68	16	11	23.5%	68.8%
	white-D + white-I	66	16	9	24.2%	56.3%
	white-D + white-C	55	12	11	21.8%	91.7%
	white-E + white-H	65	16	7	24.6%	43.8%
	white-E + white-I	65	16	9	24.6%	56.3%
	white-E + white-A	65	17	6	26.2%	35.3%
	white-F + white-A	65	16	8	24.6%	50.0%
	white-E + white-G	50	9	9	18.0%	100.0%
	white-E + white-A	55	13	13	23.6%	100.0%
	white-E + white-O	60	13	12	21.7%	92.3%
$Cas9^{H840A}$	white-K + white-S	65	17	9	26.2%	52.9%
	white-F + white-L	65	19	13	29.2%	68.4%
	white-K + white-P	55	10	7	18.2%	70.0%
	white-F + white-M	55	10	5	18.2%	50.0%
	white-S + white-N	65	18	17	27.7%	94.4%
	white-E + white-F	50	7	5	14.0%	71.4%
	white-D + white-R	68	18	11	26.5%	61.1%
	white-D + white-H	50	9	4	18.0%	44.4%
	white-Q + white-B	45	7	6	15.6%	85.7%
	white-E + white-R	45	7	6	15.6%	85.7%
	white-D + white-C	55	13	9	23.6%	69.2%
	white-E + white-H	50	10	10	20.0%	100.0%
	white-E + white-I	65	16	11	24.6%	68.8%

*The survival rate is calculated as the percentage of total G0 adults divided by embryos injected.

**The fertile rate is calculated as the percentage of fertile G0 flies divided by total G0 adults.
