# Supplementary material for: Performance of the Cas9 Nickase System in Drosophila melanogaster
Source: G3 (Bethesda). 2014 Aug 15;4(10):1955–62. doi: 10.1534/g3.114.013821 (PMC4199701; doi:10.1534/g3.114.013821)
Supplement: Supporting Information [file supp_g3.114.013821_FigureS1.pdf]

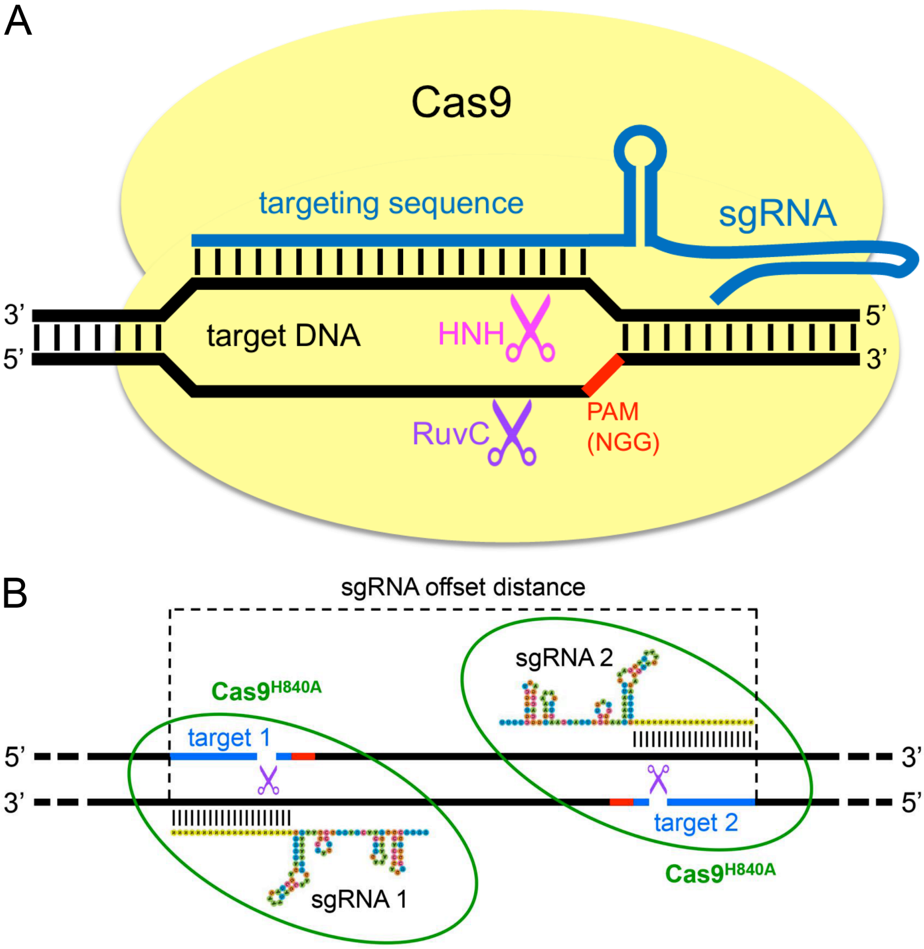

Figure S1. Models of Cas9 nuclease or Cas9^{H840A} nickase in introducing DSBs in DNA. (A) Schematic of the Cas9/sgRNA system in introducing DNA DSBs. Cas9 is shown in yellow and the chimeric sgRNA is shown in blue. The Cas9/sgRNA ribonucleoprotein recognizes the target double-stranded DNA by a 20nt complementary targeting sequence. A three nucleotide PAM sequence (NGG) is also required in the DNA for Cas9/sgRNA targeting, and is shown in red. Cas9 then introduces DSBs with the two nuclease domains. The HNH domain cuts the complementary strand to the sgRNA (magenta scissors), and the RuvC domain cuts the non-complementary strand (purple scissors). (B) Diagram showing two neighboring Cas9^{H840A}/sgRNA ribonucleoproteins targeting the fly genome. The H840A mutation converts Cas9 into a nickase that targets only the strand that is not complementary to the sgRNA. Each Cas9^{H840A} nickase is shown as a green circle. The sgRNA targets are shown in blue and the PAMs in red. The single-strand nicks are shown by the purple scissors. The offset distance is measured from the PAM-distal end of an sgRNA target to that of the other. If the PAMs are facing inward towards each other as shown in this diagram, the distance is a negative number.
