# Supplementary material for: Performance of the Cas9 Nickase System in Drosophila melanogaster
Source: G3 (Bethesda). 2014 Aug 15;4(10):1955–62. doi: 10.1534/g3.114.013821 (PMC4199701; doi:10.1534/g3.114.013821)
Supplement: Supporting Information [file supp_g3.114.013821_FigureS2.pdf]

GCTGCAAGGCGATTAAGTTGGGTAACGCCAGGGTTTTCCCAGTCACGACGTTGTAAAACGACGGCCAGTGAATTGTAATACGA
CTCACTATAGGGCGAATTGGGTACAAGCTTATTTATTTTGTTATGTTATATGTATTATATGTCAGACATAAAGAAAAGGAACA
CATCAAATGTGATAACAAAGACTAAACAAGTAATTTTATTACACCAAAACGACAAAACAGTAGGCAGAACAAACAACGCATAG
CCAAACATTGACGAATTGGATACCCTGCCGATTGTCAGACACTTTTGTTGATCAGTTTCTTGCGAATGGTCTCGTCCAGCGGT
GGAATCGCCTCGCGGGGAATCAGAAAAGTGGACAGATTGAACAGATCCAGAAACACCTTGTACCGATCACTGAAACCAAAAAA
AAACAAAGGGAGAACAGTTTGAGTTCATTGATCCCCGATATAATCACATCTGCGATGATCACCTGAGAGTGGAGCGCAGATAT
TGATAACCAGACGAGCCACCAGTGCCCAACTGTTGCGATCCAATCATGCGTTGCACCATGATCACGTGATTGTCTGCGGCGGG
AATAGAAAGTATTTGGTTAGGAAAACCAGTCTTAAACATAAGATATATTTATAAAAGAGTATCAAAGAATGCAATACTTACAT
CTCCACTTGGTTATTAACGAGTCGATGTCCATGAGCAGGGTGAGCAACTGGTGTGGTTGGCTGAACCTGGGTTCATCCCTATA
GAAGGTGATCATGATGGCTCCCTGAAGGGCACGATGGCTAAACCGGCGATCCCCACGACGCACCAGTGCATCGTGCACTGCCG
GATCAAAGATGGAGCGATACACCTCGCGTCGCTTCTCAATGTCCATGAGGCGGTAGTTTTCGCCTTCTCCACGGGCTCCTCC
ATGGCGCTCTGTACCTGCGCCTCCAGGAATCGATCGACGCTCTCCTGAAACTTGGCCCAGAAGTTGAAGCCACTCTCCTCCAG
TCCGGGCGTCCTCTCCAGCCATCGCTGCACTAGCTCCAGTAGCGAGGGATCTTTCTCCGAGTTGCGAATCGAGTTCCGCGCCT
CCTCGTCGCTAAAGACATCCGAGTACTTCTGGTTGTATCTCACCCGCTGCTCTGTCAGAACTCCCAGCTTGTTCTCGATCAAA
CGGAACTGCAGCGACTGAAAACCAGATGCGGGTGCCAGGTACTTGCGGAAGTCCATGAAGTCTAGCGGGGTCATGGTCTCCAG
AATGGGCACTTGGTCCACCAGGAGCTGTACAAAGGAAGTTATAAACGGATTTTGGTAAGAGATTCAGAAAGCACTCACTTTTA
GAATCAGAACCACTCGGTTCAGTCGCTTGACAATCTCCAGCGTCTTGGTTTCATCGATGACCTCTGCATCCAACATGTCTCGT
ATGGAGTCGAACTCAAAGATGATCTGCTTGAACCAAAGCTCGTAGGCTGTGGCGAAGGTACTTAAATGCCATTGAGTGTTGTC
ATCAAAGTTGTAAACCTACTCACCCTGGTGCGTGATGATGAACAGATGCTCATCGTGCACGGGTCGCTTGTCCTCCTCGGACA
GCATACACTGGGCATCCAGCAGTTTGTCCAGCATCAGATACTCTCCATAGATTTTGCCCACTTCCGTGGTTAATGGCACCGCC
GAATCATCGTGATCGTTTCTGTATGGGTTTGAATTGAATCGCAGAACTGAAGATCGATTGGCATTCCTGGACAGCACGTGCTG
GTGCTCACCCGTTTCCTGCATAGGGACAGCTCATGGTGCACAGCTCAGATCAGATCGTGACTCCTCGACCGGCGGATGCTGGC
GAACTGATCTCCGCCAGCGGACCGGAGATGAGACCCCAGCGAACCGATAACAGAGCGAGAGAGCTCCAGTTCCGACTGATTGC
ACAGTCGGTGATCTGGGCGATGGGCACTGCCAGATAGGCTGGGAATTATCAATCACTTGAGGTGAAAGTGCGGCGCACACAAA
TCCAAGCTTGATATCATCGATCTCGACGCTGCATCCAACGCGTTGGGAGCTCTCCGGATCAATTCGGCTTCACGTACCGTCGA
CGATGTAGGTCACGGTCTCGAAGCCGCGGTGCGGGTGCCAGGGCGTGCCCTTGGGCTCCCCGGGCGCGTACTCCACCTCACCC
ATCTGGTCCATCATGATGAACGGGTCGAGGTGGCGGTAGTTGATCCCGGCGAACGCGCGGCGCACCGGGAAGCCCTCGCCCTC
GAAACCGCTGGGCGCGGTGGTCACGGTGAGCACGGGACGTGCGACGGCGTCGGCTGGTGCGGATACGCGGGGCAGCGTCAGCG
GGTTCTCGACGGTCACGGCGGGCATGTCGACAAGCCGAATTGATCCACTAGAAGGCCTAATTCGtCTAGCAGGATCTctaggG
CTTCGACCGTTTTAACCTCGAAATATGCACATGTAAGGACGGATGTGAGCGAACGCCAGTGATGACCGGGATCAGAGGTAACC
TACCATGGTGGGGATTAGGTGACCGTTCGCAGGTAGTTTGATCGGAGCGAATGTTCGGGGGGTCTGGCGTCAGAGGCTCTAAA
CTTTATGTAATTCCTGCCGCGAAACACGCACGTATCAAGCAGTCAGCTGTTCTCTTCGTTCAGCGCGCGCCGGTGTTGCAAAA
CGAGCGCTCTTCGCCGGCGGTGGCTCGTGCGATAGTTCGTTTTGTCGGTAATCCGATGTTGCCGCGCCGATATCATGTGATGT
TGTCACAGTGCGCGAAATTCGAATGGTGGTGTGCAGTGATTGTGTTGTGACGGCGAGTGGCGCGTGTGGGTGCTTAGTTTTGG
GAGATGTTTTCGTATTTTTTTGTTGATAACTCAGGCTTTGTTGCTGTGTTGTAGTACTATTTTCCATTGCGCGGTGTCCAGCT
TTTAATTAGTGGCACATATTCTTAGCAAGTAAAAATTATTTTGCATACTATTAAATTTCTTATAAATTATTTTCTAAAATTAA
GTTTACCTTTTCAATTTTACTAAAAATATCGATATATTTATTATCGCTGGAAAACTACATTATTCCACCTCTAAGCAAGAACC
GTTAGTTGGCGCGTAGCTTTACCACAAAATTCCTGGAATTGCCGTACGCTTCGCAGTTGTTTCAAGTTGTCTAAGGGACATAC
GATTTTTTTTGCCTCTGCGTCACGATTTTAACCCAAAAGCGAGTTTAGTTACATGTACATTATTATTAGATAAAGAAGTATCG
CGAATACTTCAGTTGAATAAACTGTGCTTGGTTTTTGGGTGAGGATTTGTGGAAAGTAGAGTGCGCGATAACCGTAACTTTCG
ACCCGGATTTTCGCCtgAATTgagatctcTCTAGAggtacCGCCACCATGGCCCCAAAGAAGAAGCGGAAGGTCGGTATCCAC
GGTGTCCCAGCAGCCATGGACAAGAAGTACTCCATTGGGCTC**GCT**ATCGGCACAAACAGCGTCGGCTGGGCCGTCATTACGGA
CGAGTACAAGGTGCCGAGCAAAAAATTCAAAGTTCTGGGCAATACCGATCGCCACAGCATAAAGAAGAACCTCATTGGCGCCC
TCCTGTTCGACTCCGGGGAGACGGCCGAAGCCACGCGGCTCAAAAGAACAGCACGGCGCAGATATACCCGCAGAAAGAATCGG
ATCTGCTACCTGCAGGAGATCTTTAGTAATGAGATGGCTAAGGTGGATGACTCTTTCTTCCATAGGCTGGAGGAGTCCTTTTT
GGTGGAGGAGGATAAAAAGCACGAGCGCCACCCAATCTTTGGCAATATCGTGGACGAGGTGGCGTACCATGAAAAGTACCCAA
CCATATATCATCTGAGGAAGAAGCTTGTAGACAGTACTGATAAGGCTGACTTGCGGTTGATCTATCTCGCGCTGGCGCATATG
ATCAAATTTCGGGGACACTTCCTCATCGAGGGGGACCTGAACCCAGACAACAGCGATGTCGACAAACTCTTTATCCAACTGGT
TCAGACTTACAATCAGCTTTTCGAAGAGAACCCGATCAACGCATCCGGAGTTGACGCCAAAGCAATCCTGAGCGCTAGGCTGT
CCAAATCCCGGCGGCTCGAAAACCTCATCGCACAGCTCCCTGGGGAGAAGAAGAACGGCCTGTTTGGTAATCTTATCGCCCTG
TCACTCGGGCTGACCCCCAACTTTAAATCTAACTTCGACCTGGCCGAAGATGCCAAGCTTCAACTGAGCAAAGACACCTACGA
TGATGATCTCGACAATCTGCTGGCCCAGATCGGCGACCAGTACGCAGACCTTTTTTTGGCGGCAAAGAACCTGTCAGACGCCA
TTCTGCTGAGTGATATTCTGCGAGTGAACACGGAGATCACCAAAGCTCCGCTGAGCGCTAGTATGATCAAGCGCTATGATGAG
CACCACCAAGACTTGACTTTGCTGAAGGCCCTTGTCAGACAGCAACTGCCTGAGAAGTACAAGGAAATTTTCTTCGATCAGTC
TAAAAATGGCTACGCCGGATACATTGACGGCGGAGCAAGCCAGGAGGAATTTTACAAATTTATTAAGCCCATCTTGGAAAAAA
TGGACGGCACCGAGGAGCTGCTGGTAAAGCTTAACAGAGAAGATCTGTTGCGCAAACAGCGCACTTTCGACAATGGAAGCATC
CCCCACCAGATTCACCTGGGCGAACTGCACGCTATcCTCAGGCGGCAAGAGGATTTCTACCCCTTTTTGAAAGATAACAGGGA
AAAGATTGAGAAAATCCTCACATTTCGGATACCCTACTATGTAGGCCCCCTCGCCCGGGGAAATTCCAGATTCGCGTGGATGA

CTCGCAAATCAGAAGAGACCATCACTCCCTGGAACTTCGAGGAAGTCGTGGATAAGGGGGCCTCTGCCCAGTCCTTCATCGAA
AGGATGACTAACTTTGATAAAAATCTGCCTAACGAAAAGGTGCTTCCTAAACACTCTCTGCTGTACGAGTACTTCACAGTTTA
TAACGAGCTCACCAAGGTCAAATACGTCACAGAAGGGATGAGAAAGCCAGCATTCCTGTCTGGAGAGCAGAAGAAAGCTATCG
TGGACCTCCTCTTCAAGACGAACCGGAAAGTTACCGTGAAACAGCTCAAAGAAGACTATTTCAAAAAGATTGAATGTTTCGAC
TCTGTTGAAATCAGCGGAGTGGAGGATCGCTTCAACGCATCCCTGGGAACGTATCACGATCTCCTGAAAATCATTAAAGACAA
GGACTTCCTGGACAATGAGGAGAACGAGGACATTCTTGAGGACATTGTCCTCACCCTTACGTTGTTTGAAGATAGGGAGATGA
TTGAAGAACGCTTGAAAACTTACGCTCATCTCTTCGACGACAAAGTCATGAAACAGCTCAAGAGGCGCCGATATACAGGATGG
GGGCGGCTGTCAAGAAAACTGATCAATGGGATCCGAGACAAGCAGAGTGGAAAGACAATCCTGGATTTTCTTAAGTCCGATGG
ATTTGCCAACCGGAACTTCATGCAGTTGATCCATGATGACTCTCTCACCTTTAAGGAGGACATCCAGAAAGCACAAGTTTCTG
GCCAGGGGGACAGTCTTCACGAGCACATCGCTAATCTTGCAGGTAGCCCAGCTATCAAAAAGGGAATACTGCAGACCGTTAAG
GTCGTGGATGAACTCGTCAAAGTAATGGGAAGGCATAAGCCCGAGAATATCGTTATCGAGATGGCCCGAGAGAACCAAACTAC
CCAGAAGGGACAGAAGAACAGTAGGGAAAGGATGAAGAGGATTGAAGAGGGTATAAAAGAACTGGGGTCCCAAATCCTTAAGG
AACACCCAGTTGAAAACACCCAGCTTCAGAATGAGAAGCTCTACCTGTACTACCTGCAGAACGGCAGGGACATGTACGTGGAT
CAGGAACTGGACATCAATCGGCTCTCCGACTACGACGTGGAT**GCT**ATCGTGCCCCAGTCTTTTCTCAAAGATGATTCTATTGA
TAATAAAGTGTTGACAAGATCCGATAAAAATAGAGGGAAGAGTGATAACGTCCCCTCAGAAGAAGTTGTCAAGAAAATGAAAA
ATTATTGGCGGCAGCTGCTGAACGCCAAACTGATCACACAACGGAAGTTCGATAATCTGACTAAGGCTGAACGAGGTGGCCTG
TCTGAGTTGGATAAAGCcGGCTTCATCAAAAGGCAGCTTGTTGAGACACGCCAGATCACCAAGCACGTGGCCCAAATTCTCGA
TTCACGCATGAACACCAAGTACGATGAAAATGACAAACTGATTCGAGAGGTGAAAGTTATTACTCTGAAGTCTAAGCTGGTCT
CAGATTTCAGAAAGGACTTTCAGTTTTATAAGGTGAGAGAGATCAACAATTACCACCATGCGCATGATGCCTACCTGAATGCA
GTGGTAGGCACTGCACTTATCAAAAAATATCCCAAGCTTGAATCTGAATTTGTTTACGGAGACTATAAAGTGTACGATGTTAG
GAAAATGATCGCAAAGTCTGAGCAGGAAATAGGCAAGGCCACCGCTAAGTACTTCTTTTACAGCAATATTATGAATTTTTTCA
AGACCGAGATTACACTGGCCAATGGAGAGATTCGGAAGCGACCACTTATCGAAACAAACGGAGAAACAGGAGAAATCGTGTGG
GACAAGGGTAGGGATTTCGCGACAGTCCGGAAGGTCCTGTCCATGCCGCAGGTGAACATCGTTAAAAAGACCGAAGTACAGAC
CGGAGGCTTCTCCAAGGAAAGTATCCTCCCGAAAAGGAACAGCGACAAGCTGATCGCACGCAAAAAAGATTGGGACCCCAAGA
AATACGGCGGATTCGATTCTCCTACAGTCGCTTACAGTGTACTGGTTGTGGCCAAAGTGGAGAAAGGGAAGTCTAAAAAACTC
AAAAGCGTCAAGGAACTGCTGGGCATCACAATCATGGAGCGATCAAGCTTCGAAAAAAACCCCATCGACTTTCTCGAGGCGAA
AGGATATAAAGAGGTCAAAAAAGACCTCATCATTAAGCTTCCCAAGTACTCTCTCTTTGAGCTTGAAAACGGCCGGAAACGAA
TGCTCGCTAGTGCGGGCGAGCTGCAGAAAGGTAACGAGCTGGCACTGCCCTCTAAATACGTTAATTTCTTGTATCTGGCCAGC
CACTATGAAAAGCTCAAAGGGTCTCCCGAAGATAATGAGCAGAAGCAGCTGTTCGTGGAACAACACAAACACTACCTTGATGA
GATCATCGAGCAAATAAGCGAATTCTCCAAAAGAGTGATCCTCGCCGACGCTAACCTCGATAAGGTGCTTTCTGCTTACAATA
AGCACAGGGATAAGCCCATCAGGGAGCAGGCAGAAAACATTATCCACTTGTTTACTCTGACCAACTTGGGCGCGCCTGCAGCC
TTCAAGTACTTCGACACCACCATAGACAGAAAGCGGTACACCTCTACAAAGGAGGTCCTGGACGCCACACTGATTCATCAGTC
AATTACGGGGCTCTATGAAACAAGAATCGACCTCTCTCAGCTCGGTGGAGACAAGCGTCCTGCTGCTACTAAGAAAGCTGGTC
AAGCTAAGAAAAAGAAATAATctagcacctaggaggatccactcgAGGTTTAGAGAGGGCGAATCCAGCTCTGGAGCAGAGGC
TCTGGCAGCTTTTGCAGCGTTTATATAACATGAAATATATATACGCATTCCGATCAAAGCTGGGTTAACCAGATAGATAGATA
GTAACGTTTAAATAGCGCCTGGCGCGTTCGATTTTAAAGAGATTTAGAGCGTTATCCCGTGCCTATAGATCaTATAGTATAGA
CAACGAACGATCACTCAAATCCAAGTCAATAATTCAAGAATTTATGTCTGTTTCTGTGAAAGGGAAACTAATTTTGTTAAAGA
AGACTTACAATATCGTAATACTTGTTCAATCGTCGTGGCCGATAGAAATATCTTACAATCCGAAAGTTGATGAATGGAATTGG
TCTGCAACTGGTCGCCTTCATTTCGTAAAATGTTCGCTTGCGGCCGAAAAATTTCGATATATCTACAATTcATCTACAATCTT
TACTAAATTTTGAAAAAGGAACACTTTGAATTTCGAACTGTCAATCGTATCATTAGAATTTAATCTAAATTTAAATCTTGCTA
AAGGAAATAGCAAGGAACACTTTCGTCGTCGGCTACGCATTCATTGTAAAATTTTAAATTTTGACATTCCGCACTTTTTGATA
GATAAGCGAAGAGTATTTTTATTACATGTATCGCAAGTATTCATTTCAACACACATATCTATATATATATATATATATATATA
TATATATATATATATATATGTTATATATTTATTCAATTTTGTTTACCATTGATCAATTTTTCACACATGAAACAACCGCCAGC
ATTATATAATTTTTTTATTTTTTTAAAAAATGTGTACACATATTCTGAAAATGAAAAATTCAATGGCTCGAcTGCCAAATAAA
GAAATGGTTACAATTTAAGGAAACAAATGTCCTTCTTGCACTAGTGAGCTCCAGCTTTTGTTCCCTTTAGTGAGGGTTAATTT
CGAGCTTGGCGTAATCATGGTCATAGCTGTTTCCTGTGTGAAATTGTTATCCGCTCACAATTCCACACAACATACGAGCCGGA
AGCATAAAGTGTAAAGCCTGGGGTGCCTAATGAGTGAGCTAACTCACATTAATTGCGTTGCGCTCACTGCCCGCTTTCCAGTC
GGGAAACCTGTCGTGCCAGCTGCATTAATGAATCGGCCAACGCGCGGGGAGAGGCGGTTTGCGTATTGGGCGCTCTTCCGCTT
CCTCGCTCACTGACTCGCTGCGCTCGGTCGTTCGGCTGCGGCGAGCGGTATCAGCTCACTCAAAGGCGGTAATACGGTTATCC
ACAGAATCAGGGGATAACGCAGGAAAGAACATGTGAGCAAAAGGCCAGCAAAAGGCCAGGAACCGTAAAAAGGCCGCGTTGCT
GGCGTTTTTCCATAGGCTCCGCCCCCCTGACGAGCATCACAAAAATCGACGCTCAAGTCAGAGGTGGCGAAACCCGACAGGAC
TATAAAGATACCAGGCGTTTCCCCCTGGAAGCTCCCTCGTGCGCTCTCCTGTTCCGACCCTGCCGCTTACCGGATACCTGTCC
GCCTTTCTCCCTTCGGGAAGCGTGGCGCTTTCTCATAGCTCACGCTGTAGGTATCTCAGTTCGGTGTAGGTCGTTCGCTCCAA
GCTGGGCTGTGTGCACGAACCCCCCGTTCAGCCCGACCGCTGCGCCTTATCCGGTAACTATCGTCTTGAGTCCAACCCGGTAA
GACACGACTTATCGCCACTGGCAGCAGCCACTGGTAACAGGATTAGCAGAGCGAGGTATGTAGGCGGTGCTACAGAGTTCTTG
AAGTGGTGGCCTAACTACGGCTACACTAGAAGGACAGTATTTGGTATCTGCGCTCTGCTGAAGCCAGTTACCTTCGGAAAAAG
AGTTGGTAGCTCTTGATCCGGCAAACAAACCACCGCTGGTAGCGGTGGTTTTTTTGTTTGCAAGCAGCAGATTACGCGCAGAA
AAAAAGGATCTCAAGAAGATCCTTTGATCTTTTCTACGGGGTCTGACGCTCAGTGGAACGAAAACTCACGTTAAGGATTTTG

GTCATGAGATTATCAAAAAGGATCTTCACCTAGATCCTTTTAAATTAAAAATGAAGTTTTAAATCAATCTAAAGTATATATGA
GTAAACTTGGTCTGACAGTTACCAATGCTTAATCAGTGAGGCACCTATCTCAGCGATCTGTCTATTTCGTTCATCCATAGTTG
CCTGACTCCCCGTCGTGTAGATAACTACGATACGGGAGGGCTTACCATCTGGCCCCAGTGCTGCAATGATACCGCGAGACCCA
CGCTCACCGGCTCCAGATTTATCAGCAATAAACCAGCCAGCCGGAAGGGCCGAGCGCAGAAGTGGTCCTGCAACTTTATCCGC
CTCCATCCAGTCTATTAATTGTTGCCGGGAAGCTAGAGTAAGTAGTTCGCCAGTTAATAGTTTGCGCAACGTTGTTGCCATTG
CTACAGGCATCGTGGTGTCACGCTCGTCGTTTGGTATGGCTTCATTCAGCTCCGGTTCCCAACGATCAAGGCGAGTTACATGA
TCCCCCATGTTGTGCAAAAAAGCGGTTAGCTCCTTCGGTCCTCCGATCGTTGTCAGAAGTAAGTTGGCCGCAGTGTTATCACT
CATGGTTATGGCAGCACTGCATAATTCTCTTACTGTCATGCCATCCGTAAGATGCTTTTCTGTGACTGGTGAGTACTCAACCA
AGTCATTCTGAGAATAGTGTATGCGGCGACCGAGTTGCTCTTGCCCGGCGTCAATACGGGATAATACCGCGCCACATAGCAGA
ACTTTAAAAGTGCTCATCATTGGAAAACGTTCTTCGGGGCGAAAACTCTCAAGGATCTTACCGCTGTTGAGATCCAGTTCGAT
GTAACCCACTCGTGCACCCAACTGATCTTCAGCATCTTTTACTTTCACCAGCGTTTCTGGGTGAGCAAAAACAGGAAGGCAAA
ATGCCGCAAAAAAGGGAATAAGGGCGACACGGAAATGTTGAATACTCATACTCTTCCTTTTTCAATATTATTGAAGCATTTAT
CAGGGTTATTGTCTCATGAGCGGATACATATTTGAATGTATTTAGAAAAATAAACAAATAGGGGTTCCGCGCACATTTCCCCG
AAAAGTGCCACCTAAATTGTAAGCGTTAATATTTTGTTAAAATTCGCGTTAAATTTTTGTTAAATCAGCTCATTTTTTAACCA
ATAGGCCGAAATCGGCAAAATCCCTTATAAATCAAAAGAATAGACCGAGATAGGGTTGAGTGTTGTTCCAGTTTGGAACAAGA
GTCCACTATTAAAGAACGTGGACTCCAACGTCAAAGGGCGAAAAACCGTCTATCAGGGCGATGGCCCACTACGTGAACCATCA
CCCTAATCAAGTTTTTTGGGGTCGAGGTGCCGTAAAGCACTAAATCGGAACCCTAAAGGGAGCCCCCGATTTAGAGCTTGACG
GGGAAAGCCGGCGAACGTGGCGAGAAAGGAAGGGAAGAAAGCGAAAGGAGCGGGCGCTAGGGCGCTGGCAAGTGTAGCGGTCA
CGCTGCGCGTAACCACCACACCCGCCGCGCTTAATGCGCCGCTACAGGGCGCGTCCCATTCGCCATTCAGGCTGCGCAACTGT
TGGGAAGGGCGATCGGTGCGGGCCTCTTCGCTATTACGCCAGCTGGCGAAAGGGGGATGT

Figure S2. Full-length sequence of the *nos*-Cas9^{D10A} (or *nos*-Cas9^{H840A}) plasmid. The key components of the plasmid are color-coded. The *vermilion* reporter, magenta; *attB* site, brown; the *nos* promoter, 5'UTR, and 3'UTR, cyan; *Cas9* gene, green; the D10A mutation, red (or the H840A mutation, purple).
