# Supplementary material for: Performance of the Cas9 Nickase System in Drosophila melanogaster
Source: G3 (Bethesda). 2014 Aug 15;4(10):1955–62. doi: 10.1534/g3.114.013821 (PMC4199701; doi:10.1534/g3.114.013821)
Supplement: Supporting Information [file supp_g3.114.013821_FigureS3.pdf]

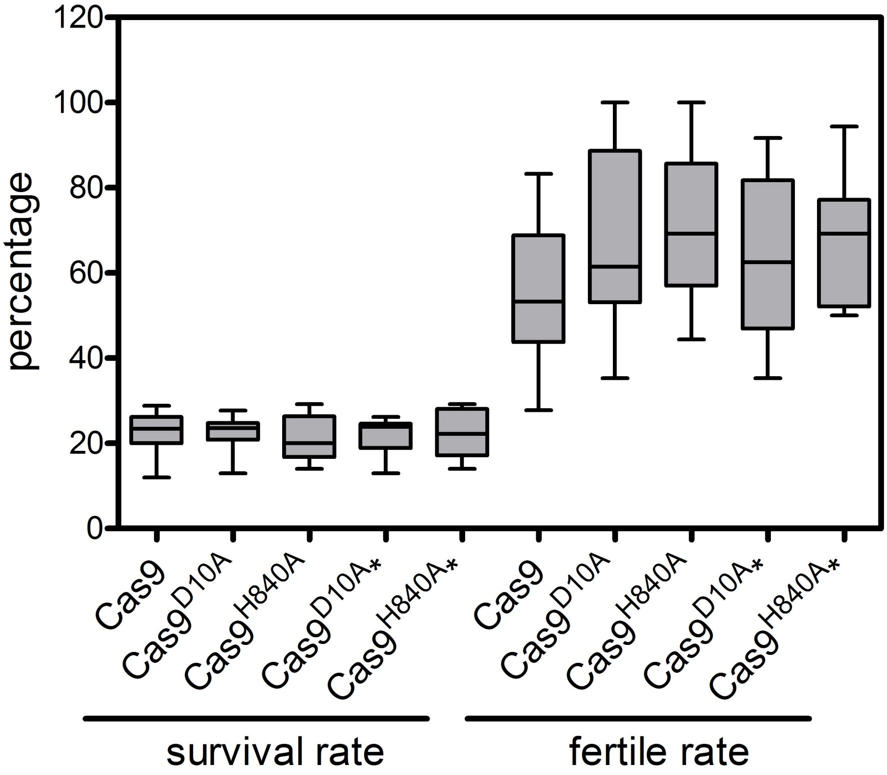

Figure S3. No significant improvement of survival and fertile G0 rates when applying Cas9 nickase. Using data from Table S3, the average survival and fertile G0 rates are calculated for Cas9 with one sgRNA (Cas9), or for Cas9 nickase with offset sgRNA pairs ($Cas9^{D10A}$ and $Cas9^{H840A}$). In addition, survival and fertile G0 rates for Cas9 nickase with paired sgRNAs that successfully generated heritable mutants are also calculated ($Cas9^{D10A*}$ and $Cas9^{H840A*}$). No significant differences are observed among five groups, for either survival rate or fertile rate.
