# Supplementary material for: Performance of the Cas9 Nickase System in Drosophila melanogaster
Source: G3 (Bethesda). 2014 Aug 15;4(10):1955–62. doi: 10.1534/g3.114.013821 (PMC4199701; doi:10.1534/g3.114.013821)
Supplement: Supporting Information [file supp_g3.114.013821_FigureS4.pdf]

NGG:PAM **N**:mismatch

CAATATGGACATCTTTGGGG**CGG** white-sgRNA-A
TA**T**TA**A**GGACATCTTTGGG**T**CGG potential off-target site A1

CAGGAGCTATTAATTCGCGG**AGG** white-sgRNA-B
CAGGAGCTATTAAT**GTC**CG**C**CGG potential off-target site B1

GCACAATATGGACATCTTTG**GGG** white-sgRNA-C
CC**TG**AA**C**ATGGACATCTTTG**CAG** potential off-target site C1

AGGTGAGGTTCTCGGCTAGT**TGG** white-sgRNA-D
A**AC**TGA**T**G**A**TCTCGGCTAGT**TGG** potential off-target site D1
GG**A**TGAGGTTCTCGGCT**G**G**C**TGG potential off-target site D2

Figure S4. No off-target effect detected using the transgenic Cas9 nickase flies to generate heritable mutations. F1 flies from three groups of successful *white* mutations using Cas9^{D10A} flies and pairs of sgRNAs are tested. Four sgRNA on-target sequences and their corresponding potential off-target sites are listed. Genomic regions that have no more than four mismatches to the on-target and a neighboring PAM are sequenced from eight F1 white-eyed flies for each group. No mutations were detected at the potential off-targets. Mismatches between the potential off-targets and the targeted region are shown in red. The PAM sequences are in bold type.
