# Supplementary material for: Performance of the Cas9 Nickase System in Drosophila melanogaster
Source: G3 (Bethesda). 2014 Aug 15;4(10):1955–62. doi: 10.1534/g3.114.013821 (PMC4199701; doi:10.1534/g3.114.013821)
Supplement: Supporting Information [file supp_g3.114.013821_FigureS5.pdf]

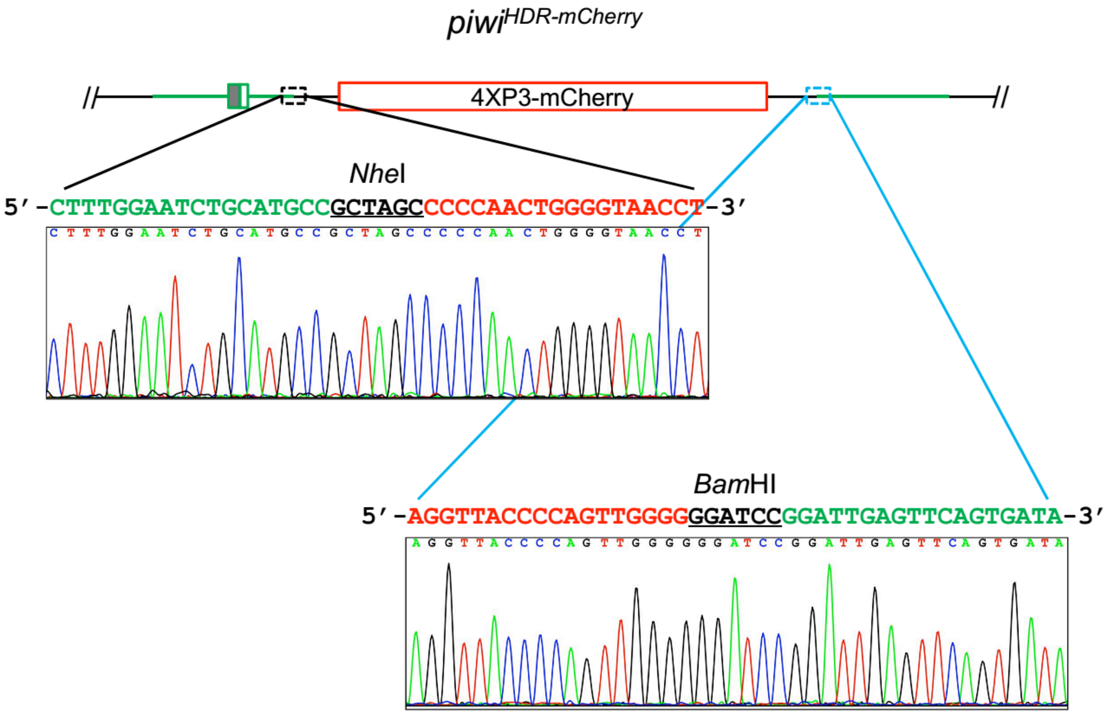

Figure S5. Representative sequencing results at the break points of the *piwi*$^{HDR\text{-}mCherry}$ mutant lines. The two boxes show parts of the sequencing results that represent the break points on the genomic DNA, with the genomic sequence in green and the 4XP3-mCherry sequence in red. Sequencing results with all six *piwi*$^{HDR\text{-}mCherry}$ lines show the same result.
