# Supplementary material for: Performance of the Cas9 Nickase System in Drosophila melanogaster
Source: G3 (Bethesda). 2014 Aug 15;4(10):1955–62. doi: 10.1534/g3.114.013821 (PMC4199701; doi:10.1534/g3.114.013821)
Supplement: Supporting Information [file supp_g3.114.013821_FigureS6.pdf]

NGG: PAM **N**: mismatch

piwi-HDR-sgRNA-left

CGGACATGACCAAATGGACC**CGG**

CGGACATGACCAAAT**T**GAC**T****CGG** potential off-target site L1

CGGACATGACCA**G**AT**C**GACC**GGG** potential off-target site L2

CGGACATGACCAAAT**C**GA**GT****CGG** potential off-target site L3

CGGACATGACCA**G**AT**C**GAC**T****AGG** potential off-target site L4

CGGACATGACCA**G**AT**C**GAC**T****CAG** potential off-target site L5

piwi-HDR-sgRNA-right

TATCGATTGCAAGTTGGGGT**AGG**

TATCGATTGCAA**A**TTG**T**G**CC****TGG** potential off-target site R1

TATCG**T**TTGCAAGTTG**CTA**T**CAG** potential off-target site R2

Figure S6. No off-target effect detected using the CRISPR system to generate *piwi* mutations. Flies from six successful *piwi*$^{HDR\text{-}mCherry}$ mutation strains are tested. The two sgRNA on-target sequences and their corresponding potential off-target sites are listed. Genomic regions that have no more than four mismatches to the on-target and a neighboring PAM are sequenced. No mutations were detected at the potential off-targets. Mismatches between the potential off-targets and the targeted region are shown in red. The PAM sequences are in bold type.
