# Supplementary material for: Performance of the Cas9 Nickase System in Drosophila melanogaster
Source: G3 (Bethesda). 2014 Aug 15;4(10):1955–62. doi: 10.1534/g3.114.013821 (PMC4199701; doi:10.1534/g3.114.013821)
Supplement: Supporting Information [file supp_g3.114.013821_TableS1.pdf]

Table S1. Sequences of the oligonucleotides for mutation detection and vector construction in this study.

Use	Name	Sequence
Indel mutation detection	White-F	TATCATTGCAGGGTGACAGCG
	White-R	GGCATTCAGCAGGGTCGTC
Cas9 nickase mutagenesis	Cas9-D10A-F	CTCCATTGGGCTCGCTATCGGCACAAACA
	Cas9-D10A-R	TGTTTGTGCCGATAGCGAGCCCAATGGAG
	Cas9-H840A-F	TACGACGTGGATGCTATCGTGCCCCAG
	Cas9-H840A-R	CTGGGGCACGATAGCATCCACGTCGTA
piwi-4XP3-mCherry donor template homologous arms	piwi-L-F	GGCCTAGGTGCGGTATTTAAGGCTCTTGC
	piwi-L-R	CTAGCTAGCGGCATGCAGATTCCAAAGAC
	piwi-R-F	CGGGATCCGGATTGAGTTCAGTGATAATATCG
	piwi-R-R	GGACTAGTCGCAACTGAGCAATACTTTCCAC
piwi-4XP3-mCherry donor template mCherry marker	mCherry-F	CCGCTCGAGATGGTGAGCAAGGGCGAGGA
	mCherry-R	CGGGGTACCTTACTTGTACAGCTCGTCCATGCC
	SV40-F	CGGGGTACCGATCTTTGTGAAGGAACC
	SV40-R	CGGGATATCACTAGACTGGAACCAGACATG
$piwi^{HDR\text{-}mCherry}$ confirmation	primer 1	TTACTTGCATAGCAGGGTGGCTG
	primer 2	CTCCGGCGCTCGTCTAGTGTCG
	primer 3	CACACCTCCCCCTGAACCTG
	primer 4	GCCTCCACCAGTTCCAGACG
