# Supplementary material for: Performance of the Cas9 Nickase System in Drosophila melanogaster
Source: G3 (Bethesda). 2014 Aug 15;4(10):1955–62. doi: 10.1534/g3.114.013821 (PMC4199701; doi:10.1534/g3.114.013821)
Supplement: Supporting Information [file supp_g3.114.013821_TableS2.pdf]

Table S2. Sequences of the oligonucleotides for off-target effect analysis.

primers	Sequence (5' to 3')
white-OTE-A1-F	CGACACCAGCTATGGTTCGG
white-OTE-A1-R	CTCGGTATGGACCTAGAGGC
white-OTE-B1-F	AACAAACAACCGCTCAACCC
white-OTE-B1-R	GAATCGGGCGTAGAAGACAG
white-OTE-C1-F	TGTTCGTCGTCCTCTTCCTCC
white-OTE-C1-R	AGGAGCACAAGGCGAAGCAC
white-OTE-D1-F	ACCAAGGGTGGATAGGTGCG
white-OTE-D1-R	GGGTTGAAACTTCCTACTTGCC
white-OTE-D2-F	GATCTTTGGCTTTCGTTCAGG
white-OTE-D2-R	TTGAACCGCCCAACTGCTCC
piwi-OTE-L1-F	GAACCTGAATGCCTAATCTC
piwi-OTE-L1-R	CGATTTGACTTTGGGCAAGA
piwi-OTE-L2-F	CAACTGACCTCTTAGCCCTCG
piwi-OTE-L2-R	TAGAGCGAGATTTCCTTGAGC
piwi-OTE-L3-F	ACTGGGACCTACGATTGCC
piwi-OTE-L3-R	AAGCGAGACCGCACTACCG
piwi-OTE-L4-F	GGAGGGTAAAGGGCACGAAA
piwi-OTE-L4-F	CAATGAGTTTGCCCTTGACG
piwi-OTE-L5-F	CGATAGAAATGGGCAAAGCAAC
piwi-OTE-L5-R	GTGGAGGTGGTATTTATTGCCC
piwi-OTE-R1-F	GTCATTAAGTCTGGCCTGTGGG
piwi-OTE-R1-R	AGGGCTCAATGTTGAACGAAGC
piwi-OTE-R2-F	CATCGCATCCATGATTTCCTC
piwi-OTE-R2-R	AGGCGCAAAGTATTTCACCC
